# Ammonia reduces glutamine synthetase expression in astrocytes via activation of Hippo-YAP signaling pathways

**DOI:** 10.1038/s42003-025-09191-5

**Published:** 2025-12-13

**Authors:** Yusuke Nasu, Sari Kishikawa, Mamiko Imai, Nozomi Yokoyama, Izumi Iida, Koichi Tabeta, Miho Terunuma

**Affiliations:** 1 Division of Oral Biochemistry, Faculty of Dentistry & Graduate School of Medical and Dental Sciences, Niigata University, Niigata, Japan; 2 Division of Periodontology, Faculty of Dentistry & Graduate School of Medical and Dental Sciences, Niigata University, Niigata, Japan; 3 Department of Life Sciences, Graduate School of Arts and Sciences, The University of Tokyo, Tokyo, Japan

**Keywords:** Astrocyte, Epilepsy

## Abstract

Glutamine synthetase (GS) expressed in astrocytes plays an important role in maintaining the amount of extracellular glutamate and ammonia in the brain. Deficiency of GS is associated with several neurological disorders including epilepsy, but the molecular mechanism underlying the control of GS expression and the mechanism of GS reduction in diseased brain remain elusive. Here we show that in astrocytes, GS level is regulated by a transcription coactivator YAP via two signaling pathways, the Hippo and Wnt/β-catenin pathways. But when the extracellular glutamate and ammonia are increased, ammonia inhibits YAP nuclear localization through Hippo pathways and reduces GS. Using rodent models of epilepsy, we found that the induction of YAP nuclear translocation with Hippo kinase inhibitor XMU-MP-1 suppresses GS downregulation and protects neurons against cell death. Our work identifies that YAP may become an effective target for diseases in which astrocytic GS is reduced due to elevated ammonia.

## Abstract (web summary)

Glutamine synthetase expression in astrocytes is regulated by YAP through Hippo pathways to metabolize ammonia and glutamate in the brain, and temporarily turning off this pathway during seizure may be an effective treatment for epilepsy.

## Introduction

In brain, astrocytes that are responsible for regulating neural functions by synthesizing and releasing various chemicals called gliotransmitters, coordinate the surrounding neural activities^1^. One of their important functions is the metabolism of two neurotoxic agents: ammonia and glutamate, using the enzyme called glutamine synthetase (GS). GS is exclusively expressed in astrocytes, and for this reason, it is often used as a reliable cell marker for astrocytes. Astrocytes take up ammonia from the circulation as well as an excessive extracellular glutamate released from the neurons and convert them to glutamine by the enzymatic activation of GS. Glutamine becomes a source of glutamate and gamma aminobutyric acid (GABA), two important neurotransmitters, thus astrocytic GS is an essential enzyme in the brain. Previous studies have reported that in epileptic patients’ brain, expression of astrocytic GS is reduced, while blood ammonia and brain glutamate are elevated^2^. GS reduction has also been associated with Alzheimer’s disease, traumatic brain injury, and Parkinson’s disease^3,4^. However, the molecular mechanism regulating astrocytic GS expression and the mechanism of GS reduction in these disorders remains unclear.

Outside of the brain, GS is mainly expressed in the liver and detoxifies ammonia released into the systemic circulation^5,6^. GS incorporates ammonia into glutamine by selectively expressing in hepatocytes, which becomes a fundamental source of nitrogen for amino acids and nucleotides^7,8^. In the zebrafish liver cancer model, YAP reprograms glutamine metabolism by enhancing de novo nucleotide biosynthesis and regulates GS expression to induce cell proliferation^9^. YAP is a major transcriptional cofactor in the Hippo pathway, which has appeared as an evolutionarily conserved master regulator of cell proliferation and organ growth^10^. It also links to the Wnt/β-catenin signaling pathway, which determines cell fate, proliferation, and migration^11^. Previous studies on astrocytes have shown that YAP plays important roles in neural stem cell differentiation and proliferation^12^. Astrocytic YAP also contributes to the pathology of spinal cord injury^13^, Alzheimer’s disease^14^, and multiple sclerosis^15^. However, the relationship between YAP and GS in astrocytes have not yet studied.

Here, we report that in astrocytes, a potent transcription coactivator YAP regulates the expression of GS through two upstream signaling pathways, the Hippo pathways and Wnt/β-catenin pathways. While, when the amount of glutamate and ammonia are increased in the brain, conditions that are found in neurological disorders such as epilepsy and Alzheimer’s disease, ammonia inhibits the nuclear localization of YAP in astrocytes through activation of Hippo signaling pathway and reduces GS mRNA and protein levels. The pharmacological induction of YAP nuclear translocation with Hippo pathway kinase MST1/2 inhibitor XMU-MP-1 suppressed the reduction of GS in astrocytes occurred by the elevation of extracellular ammonia and glutamate. Finally, using rodent models of epilepsy in which astrocytic GS is reduced, we found that systemic administration of XMU-MP-1 elevates the expression of YAP in astrocyte nuclei, upregulates astrocytic GS expression, and protects neurons against cell death. Our work identifies that YAP may become an effective target for diseases in which astrocytic GS expression is reduced due to elevated ammonia.

## Results

### Glutamate and ammonium chloride co-treatment alters GS expression in primary cultured astrocytes

Glutamate and ammonium chloride (NH_4_Cl), two chemicals that become a source of glutamine, were added into rat primary cortical astrocyte culture medium and the expression of GS was examined. The level of GS was significantly increased 3 h after the treatment (Supplementary Fig. 1a, d, e). This was also evident in the single treatment with glutamate (Supplementary Fig. 1b). In contrast, NH_4_Cl did not increase GS expression by itself, but the reduction was observed 6 h after the treatment (Supplementary Fig. 1c). Of note, the intracellular glutamine but not glutamate was significantly increased 3 h after the glutamate and NH_4_Cl co-treatment, suggesting that astrocytes elevated the expression of GS to accelerate the production of glutamine via condensation of ammonia with glutamate (Supplementary Fig. 1f, g).

**Fig. 1:**
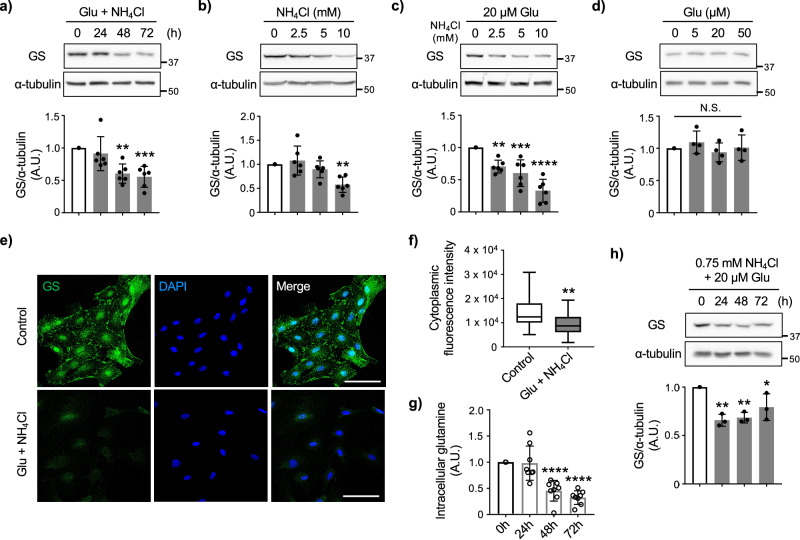
Prolonged NH_4_Cl and glutamate/NH_4_Cl treatments reduce the expression of GS in cultured astrocytes. **a** Representative western blots of GS and α-tubulin after 20 μM glutamate and 10 mM NH_4_Cl co-treatments (Glu + NH_4_Cl) for 0–72 h in cultured astrocytes. Graph shows quantification of GS normalized to α-tubulin for each experiment and expressed as percent change versus 0 h condition. n = 6 biologically independent samples. One-way ANOVA followed by Dunnett’s multiple comparisons test, **p < 0.01, ***p < 0.005. **b** Representative western blots of GS and α-tubulin at various dose of NH_4_Cl treatments for 72 h in cultured astrocytes. Graph shows quantification of GS normalized to α-tubulin for each experiment and expressed as percent change versus 0 mM condition. n = 6 biologically independent samples. One-way ANOVA followed by Dunnett’s multiple comparisons test, **p < 0.01. **c** Representative western blots of GS and α-tubulin after 72 h of glutamate (20 μM) and NH_4_Cl (0–10 mM) co-treatment in cultured astrocytes. Graph shows quantification of GS. n = 6 biologically independent samples. One-way ANOVA followed by Dunnett’s multiple comparisons test, **p < 0.01, ***p < 0.005, ****p < 0.001. **d** Representative western blots of GS and α-tubulin at various dose of glutamate stimulation for 72 h in cultured astrocytes. Graph shows quantification of GS. n = 4 biologically independent samples. One-way ANOVA followed by Dunnett’s multiple comparisons test, N.S. = not significant. **e** Representative images of immunofluorescence staining for GS (green) in cultured astrocytes with/without glutamate + NH_4_Cl treatment for 72 h. Nucleus was identified by DAPI (blue) staining. n = 3 biologically independent samples. Scale bar 100 μm. **f** Quantification of cytoplasmic GS immunostaining in astrocytes with/without glutamate + NH_4_Cl treatment for 72 h. Boxplot shows the minimum/maximum value and the median. Control: n = 29 cells, Glu + NH_4_Cl: n = 30 cells. Mann-Whitney U test, **p < 0.01. **g** Measurement of intracellular glutamine after glutamate + NH_4_Cl treatment in cultured astrocytes. n = 8 biologically independent samples. One-way ANOVA followed by Dunnett’s multiple comparisons test, ****p < 0.001. **h** Representative western blots of GS and α-tubulin after 0.75 mM NH_4_Cl and 20 μM glutamate co-treatment in cultured astrocytes. Graph shows quantification of GS normalized to α-tubulin for each experiment and expressed as percent change versus 0 h condition. n = 3 biologically independent samples. One-way ANOVA followed by Dunnett’s multiple comparisons test, *p < 0.05, **p < 0.001.

We then examined the expression of GS by the prolonged glutamate and NH_4_Cl co-treatments. Contrary to short-time treatments, the levels of GS were gradually decreased in a time-dependent manner (Fig. 1a). Along with reduced astrocytic GS expression, the decline in intracellular glutamine levels was observed (Fig. 1g). To identify which of the two extracellular compounds glutamate or NH_4_Cl is the cause of GS downregulation, individual treatments were performed. GS was reduced by the application of NH_4_Cl in a dose dependent manner (Fig. 1b), and further reduction was observed when glutamate was co-applied with NH_4_Cl (Fig. 1c), while glutamate itself did not affect GS expression (Fig. 1d). Immunocytochemistry also confirmed GS reduction by the prolonged glutamate and NH_4_Cl co-treatments (Fig. 1e, f ). Since 10 mM is the concentration of NH_4_Cl that has been used to study hyperammonemia in vitro^16^, we tested if lower concentration of NH_4_Cl is enough to reduce GS levels. We found that 0.75 mM NH_4_Cl together with glutamate significantly reduce GS expression (Fig. 1h). These results suggested that prolonged stimulation of astrocytes with glutamate and NH_4_Cl reduces GS expression. Additionally, we repeated glutamate and NH_4_Cl co-treatments in rat primary hippocampal astrocyte culture and observed similar time-dependent reduction in GS expression, suggesting that GS in CNS astrocytes is regulated by the extracellular concentration of ammonia and glutamate (Supplementary Fig. 2).

### Downregulation of GS is not due to protein degradation but rather the inhibition of protein synthesis

Since NH_4_Cl has previously been reported to induce autophagy in mouse embryo fibroblasts and tumor cells^17–19^, we wondered if GS downregulation in astrocytes upon glutamate and NH_4_Cl co-treatment was due to protein degradation. Glutamate and NH_4_Cl co-stimulation increased the ratio of LC3-II/LC3-I in a time-dependent manner (Supplementary Fig. 3a), suggesting that NH_4_Cl induced autophagy in astrocytes. We then investigated whether the autophagy inhibitor blocks GS reduction. Leupeptin that inhibits proteases within lysosomes and autophagosomes^20^ was applied with glutamate and NH_4_Cl, but the expression of GS was still reduced (Supplementary Fig. 3b). We then tested MG-132, a well-known proteasome inhibitor^21^, together with glutamate and NH_4_Cl. However, we found that the level of GS did not recover (Supplementary Fig. 3c). Importantly, a half-life of GS was not altered by the glutamate and NH_4_Cl co-treatments (Supplementary Fig. 3d, e). We thus examined the expression of GS mRNA (*glul*) to determine if protein synthesis is affected by the glutamate and NH_4_Cl co-treatments. The NH_4_Cl alone reduced *glul* only at 10 mM but co-treatment with glutamate reduced *glul* even at 2.5 mM concentration (Supplementary Fig. 3f, g). These alteration in *glul* matched to the results on GS protein levels (Fig. 1) suggesting that prolonged glutamate and ammonia stimulation inhibited GS synthesis.

### YAP is the regulator of GS level in astrocytes

**Fig. 2:**
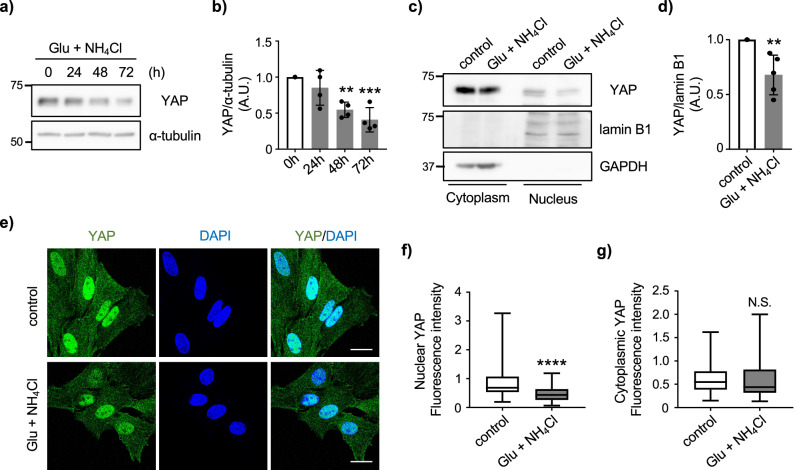
Prolonged glutamate/NH_4_Cl treatment reduces YAP nuclear localization. **a** Representative western blots of YAP and α-tubulin after 20 μM glutamate and 10 mM NH_4_Cl co-treatment (Glu + NH_4_Cl) in cultured astrocytes. **b** Quantification of GS. n = 4 biologically independent samples. One-way ANOVA followed by Dunnett’s multiple comparisons test, **p < 0.01; ***p < 0.005. **c** Subcellular fractionation of control and glutamate/NH_4_Cl (72 h)-treated astrocytes. Expression of YAP, lamin B1 (nuclear marker) and GAPDH (cytoplasmic marker) in nuclear and cytoplasmic fractions are shown. **d** Quantification of YAP in nuclear fraction. n = 5 biologically independent samples. Mann-Whitney U test, **p < 0.01. **e** Representative images of immunofluorescence staining for YAP (green) in cultured astrocytes with/without glutamate + NH_4_Cl treatment for 72 h. Nucleus was identified by DAPI (blue) staining. n = 3 biologically independent samples. Scale bar 20 μm. Quantification of YAP intensity in the nucleus (**f **) and the cytoplasm (**g**). Boxplot shows the minimum/maximum value and the median. Control n = 65 cells, glutamate and NH_4_Cl n = 67 cells. Mann-Whitney U test, ****p < 0.0001. N.S. = not significant.

YAP has been suggested as a regulator of glutamine metabolism in hepatic cancer cells to induce cell proliferation^9^. Since we found that the co-treatment of glutamate and NH_4_Cl reduced astrocyte proliferation without inducing cell death (Supplementary Fig. 4), we wondered if YAP is a regulator of GS in astrocytes. To investigate the link between YAP and GS in astrocytes, we first tested if glutamate and NH_4_Cl co-treatment reduces total level of YAP. We found that YAP expression is reduced over the course of treatments (Fig. 2a, b). Subcellular fractionation assay and immunocytochemistry confirmed that reduced expression of YAP occurs in the nucleus (Fig. 2c–g).

**Fig. 3:**
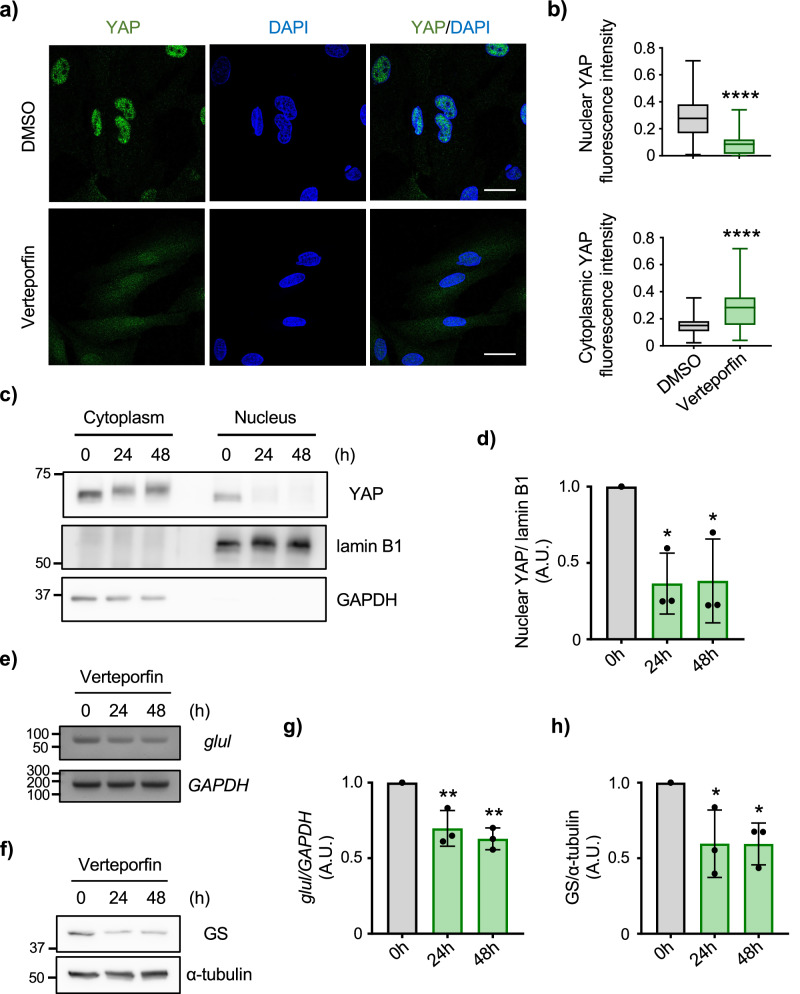
Localization of YAP influences the level of GS in astrocytes. **a** Representative images of immunofluorescence staining for YAP (green) in cultured astrocytes with/without 1 μM verteporfin for 48 h. Nucleus was identified by DAPI (blue) staining. n = 3 biologically independent samples. Scale bar 20 μm. **b** Quantification of YAP intensity in the nucleus (top) and the cytoplasm (bottom). Boxplot shows the minimum/maximum value and the median. DMSO n = 79 cells, verteporfin n = 73 cells. Mann-Whitney U test, ****p < 0.0001. **c** Subcellular fractionation of astrocytes treated with 1 μM verteporfin for various time points. Expression of YAP, lamin B1 (nuclear marker) and GAPDH (cytoplasmic marker) in nuclear and cytoplasmic fractions are shown. **d** Quantification of YAP in nuclear fraction. Graph shows quantification of YAP normalized to lamin B1 for each experiment and expressed as percent change versus 0 h condition. n = 3 biologically independent cultures. One-way ANOVA followed by Dunnett’s multiple comparisons test, *p < 0.05. **e** Representative image of RT-PCR of GS mRNA (*glul*) and *GAPDH* examining the effect of 1 μM verteporfin on GS mRNA in cultured astrocytes. Markers are shown (bp). **f** Representative western blots of GS and α-tubulin in 1 μM verteporfin-treated cultured astrocytes. **g** Graph shows quantification of GS mRNA. Expression of *glul* was normalized to *GAPDH* for each experiment and expressed as percent change versus 0 h condition. n = 3 biologically independent samples. One-way ANOVA followed by Dunnett’s multiple comparisons test, **p < 0.01. **h** Graph shows quantification of GS normalized to α-tubulin. n = 3 biologically independent samples. One-way ANOVA followed by Dunnett’s multiple comparisons test, *p < 0.05.

**Fig. 4:**
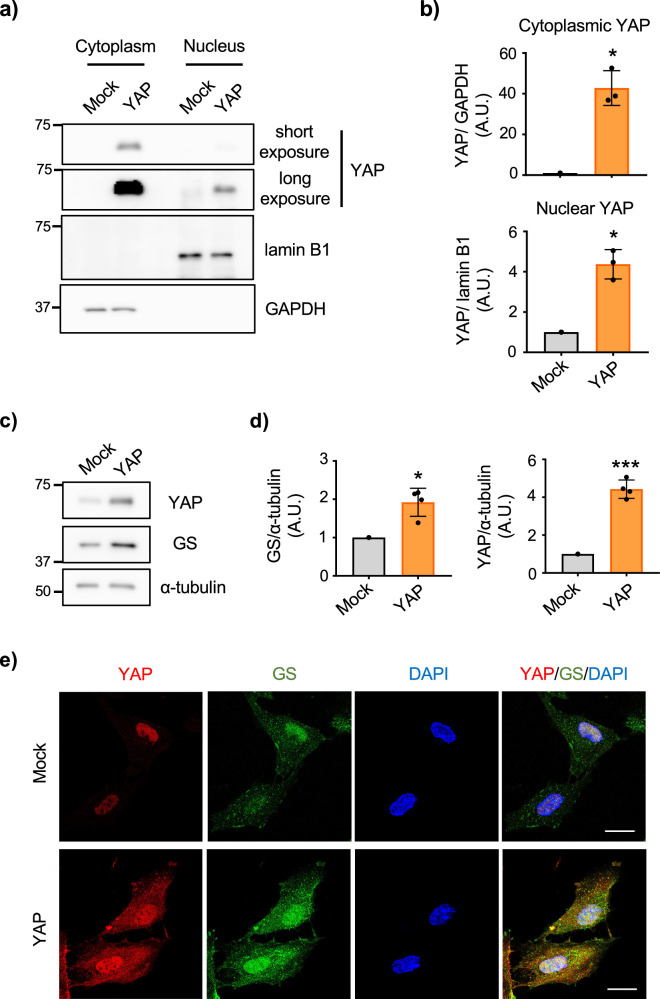
Overexpression of YAP increases GS levels in astrocytes. **a** Representative western blots of YAP and α-tubulin in the subcellular fractionation of astrocytes overexpressed with YAP. **b** Quantification of YAP in cytoplasmic (top) and nuclear (bottom) fractions prepared from astrocytes overexpressed with YAP. Bands were normalized to subcellular markers for each experiment and expressed as percent change versus mock transfected cells. n = 3 biologically independent samples. Paired *t*-test, *p < 0.05. **c** Representative western blots of YAP, GS and α-tubulin in astrocytes overexpressed with YAP. **d** Graphs show quantification of GS (left) and YAP (right) normalized to α-tubulin. n = 4 biologically independent samples. Paired *t*-test, *p < 0.05, ***p < 0.001. **e** Representative images of immunofluorescence staining for YAP (red) and GS (green) in astrocytes overexpressed with YAP. Nucleus was identified by DAPI (blue) staining. n = 4 biologically independent samples. Scale bar 20 μm.

YAP promotes transcriptional activation by its localization into the nucleus^22^. We thus used verteporfin, an inhibitor of YAP nuclear translocation, and tested whether the removal of YAP from the nucleus alters GS expression in astrocytes. By performing immunocytochemistry and subcellular fractionation, we confirmed that verteporfin reduced YAP nuclear localization (Fig. 3a–d). Along with the inhibition of YAP localization into the nucleus by verteporfin, both GS mRNA and protein were reduced (Fig. 3e–h). The association between YAP and GS was further examined by the overexpression of YAP in cultured astrocytes. Astrocytes that were overexpressed with YAP not only increased the levels of cytoplasmic YAP but also nuclear YAP (Fig. 4a, b), and GS upregulation was identified (Fig. 4c–e). Together, these results indicated that YAP directly involves in the expression of GS in astrocytes.

**Fig. 5:**
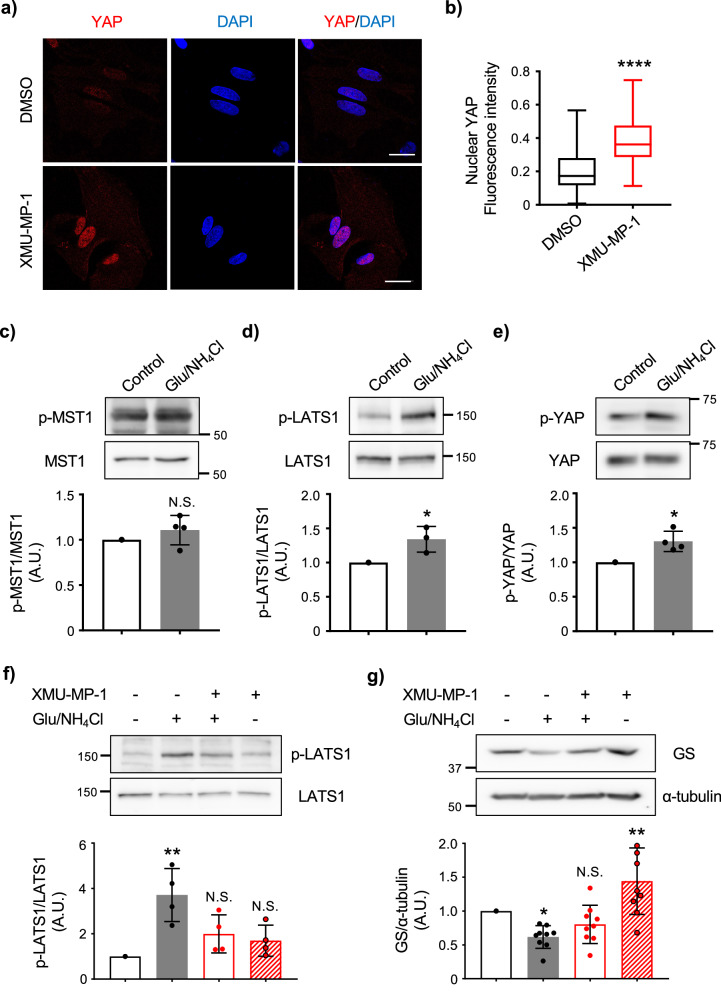
The Hippo signaling pathway act on GS expression and its inhibition recovers YAP expression reduced by glutamate/NH_4_Cl co-treatments. **a** Representative images of immunofluorescence staining for YAP in astrocytes treated with 3 μM XMU-MP-1 for 48 h. Expression of YAP is shown. Nucleus was identified by DAPI (blue) staining. n = 3 biologically independent samples. Scale bar 20 μm. **b** Quantification of YAP intensity in the nucleus. Boxplot shows the minimum/maximum value and the median. DMSO n = 85 cells, XMU-MP-1 n = 77 cells. Mann-Whitney U test, ****p < 0.0001. **c** Representative western blots of p-MST1 and MST in astrocytes treated with glutamate (20 μM) + NH_4_Cl (10 mM) for 72 h. Graph shows quantification of MST1 phosphorylation expressed as percent change versus control. n = 4 biologically independent samples. Paired *t*-test, N.S. = not significant. **d** Representative western blots of p-LATS1 and LATS1 in astrocytes treated with glutamate (20 μM) + NH_4_Cl (10 mM) for 72 h. Graph shows quantification of LATS1 phosphorylation expressed as percent change versus control. n = 3 biologically independent samples. Paired *t*-test, *p < 0.05. **e** Representative western blots of p-YAP (Ser127) and YAP in cytoplasmic fractions of astrocytes treated with glutamate (20 μM) + NH_4_Cl (10 mM) for 72 h. Graph shows quantification of YAP phosphorylation expressed as percent change versus control. n = 4 biologically independent samples. Paired *t*-test, *p < 0.05. **f** Representative western blots of p-LATS1 and LATS1 in astrocytes treated with glutamate (20 μM) + NH_4_Cl (10 mM) and/or XMU-MP-1 (3 μM) for 48 h. Graph shows quantification of LATS phosphorylation for each experiment and expressed as percent change versus XMU-MP-1(-)/Glu+NH_4_Cl(-) condition. n = 4 biologically independent samples. One-way ANOVA followed by Dunnett’s multiple comparisons test, **p < 0.01. N.S. = not significant. **g** Representative western blots of GS and α-tubulin in astrocytes treated with glutamate (20 μM) + NH_4_Cl (10 mM) and/or XMU-MP-1 (3 μM) for 48 h. Graph shows quantification of GS for each experiment and expressed as percent change versus XMU-MP-1(-)/Glu+NH_4_Cl(-) condition. n = 9 biologically independent samples. One-way ANOVA followed by Dunnett’s multiple comparisons test, *p < 0.05, **p < 0.01. N.S. = not significant.

YAP is the downstream effector of Hippo pathway which acts as a transcriptional coactivator regulating organ size and stem cell property by governing cell proliferation and apoptosis^23^. The transcriptional activity of YAP through Hippo pathway is regulated by upstream kinases MST1/2 and large tumor suppressor kinases 1/2 (LATS1/2), then the phosphorylation of YAP Serine 127 (Ser127) by LATS1/2 leads to its retention in the cytoplasm and is targeted for proteasomal degradation^24^. To determine if Hippo-YAP-mediated signaling pathways undoubtedly regulate GS expression, we treated cultured astrocytes with XMU-MP-1, a potent and selective inhibitor of the Hippo pathway kinases MST1/2. We found that XMU-MP-1 induces YAP nuclear translocation (Fig. 5a, b). We therefore treated astrocytes with glutamate and NH_4_Cl and examined the phosphorylation of MST1, LATS1 and YAP to observe if glutamate and NH_4_Cl can activate Hippo signaling pathway. We found that glutamate and NH_4_Cl co-stimulation increase the phosphorylation of LATS1 (Fig. 5d) and YAP (Fig. 5e), but no change was observed in the phosphorylation of MST1 (Fig. 5c), an upstream kinase of LATS1. Furthermore, an increased phosphorylation of LATS1 induced by the co-treatment of glutamate with NH₄Cl was suppressed by XMU-MP-1 (Fig. 5f ). In accordance with the change in LATS1 phosphorylation, the reduction in GS expression caused by glutamate/NH₄Cl co-treatment was successfully restored by XMU-MP-1 (Fig. 5g). These results suggested that the extracellular elevation of glutamate and NH_4_Cl activates LATS1, promotes YAP proteasomal degradation and inhibits GS expression.

Since YAP also acts as a transcriptional regulator of the Wnt signaling pathway by binding to β-catenin and regulates cell proliferation^11^, we tested this pathway on GS expression using FH535, an inhibitor of β-catenin/co-factors interaction in the nucleus. We found that astrocytic GS expression is inhibited when β-catenin signaling pathway is blocked (Supplementary Fig. 5a). However, the activation of Wnt/β-catenin signaling pathway by a Wnt analog SKL2001 did not recover GS expression reduced by NH_4_Cl and glutamate co-treatment (Supplementary Fig. 5b). In addition, we found that SKL2001 reduces GS expression in astrocytes by itself (Supplementary Fig. 5b). Together, these results suggested that in astrocytes, both the endogenous Hippo signaling pathway and Wnt/β-catenin signaling pathway regulate YAP expression, which resulted in the modulation of GS. But in NH_4_Cl/glutamate co-treatment-induced GS downregulation, which is a pathological condition in brain, astrocytes require YAP translocation by inhibiting Hippo pathway to promote GS expression.

**Fig. 6:**
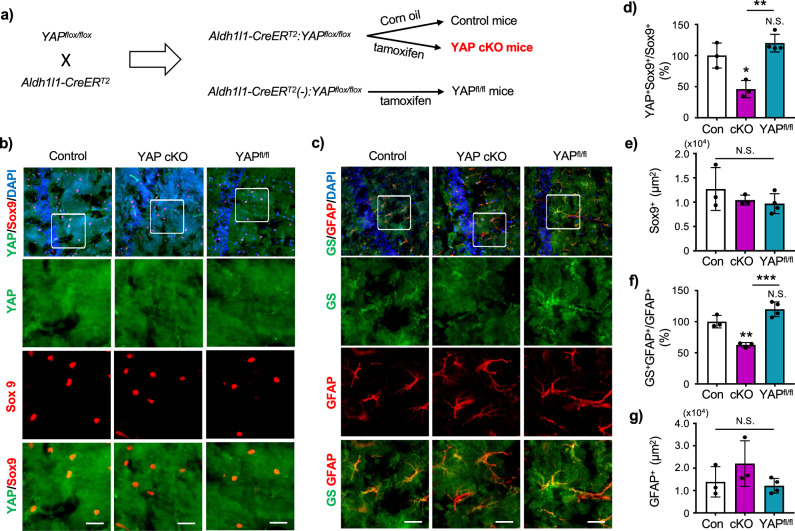
Astrocyte specific YAP conditional knockout mice reduce the expression of GS in astrocytes. **a** Scheme demonstrating the production of animals for the analysis of YAP depletion in astrocytes following tamoxifen injection. **b** Visualization of hippocampal CA1 astrocytic nuclei and YAP in YAP cKO mice and control mice. Expression of Sox9 (red), as a marker of astrocyte nucleus, and YAP (green) are shown. Nucleus was identified by DAPI (blue) staining. Scale bar 20 μm. **c** Visualization of hippocampal CA1 astrocytes in YAP cKO mice and control mice. Expression of GFAP (red) and GS (green) are shown. Nucleus was identified by DAPI (blue) staining. Scale bar 20 μm. **d** Quantification of YAP colocalization with astrocyte nuclei marker Sox9. **e** Quantification of Sox9 positive area in hippocampal CA1 in mice. **f** Quantification of GS colocalization with GFAP in hippocampal CA1 in mice. **g** Quantification of GFAP positive area in hippocampal CA1 in mice. Control: n = 3, YAP cKO: n = 3, YAP^fl/fl^: n = 4 animals per group. One-way ANOVA followed by Tukey’s multiple comparisons test, *p < 0.05, **p < 0.01, ***p < 0.001. N.S. = not significant.

To confirm that YAP regulates GS expression in mouse brain astrocytes, we crossed YAP^flox/flox^ mice^25^ and Aldh1l1-CreER^T2^ mice^26^ to generate Aldh1l1-CreER^T2^:YAP^flox/flox^ mice, and selectively knocked out YAP in astrocytes upon tamoxifen administration (Fig. 6a). In tamoxifen-treated Aldh1l1-CreER^T2^:YAP^flox/flox^ (YAP cKO) mice, co-localization of YAP with the astrocyte-specific nuclear marker Sox9^27^, was significantly reduced in the hippocampal CA1 region compared to their negative controls, confirming the effective deletion of YAP in astrocytes (Fig. 6b, d). No change in Sox9+ area was observed by YAP deletion (Fig. 6e). In the same region, GS expression in glial fibrillary acidic protein (GFAP)-positive astrocytes in YAP cKO mice were reduced although the GFAP immunoreactivity remained unchanged (Fig. 6c, f, g). These data support our findings that YAP functions as one of the regulatory factors for GS in astrocytes.

### XMU-MP-1 recovers astrocytic GS expression in epileptic brain and inhibits neuronal death

**Fig. 7:**
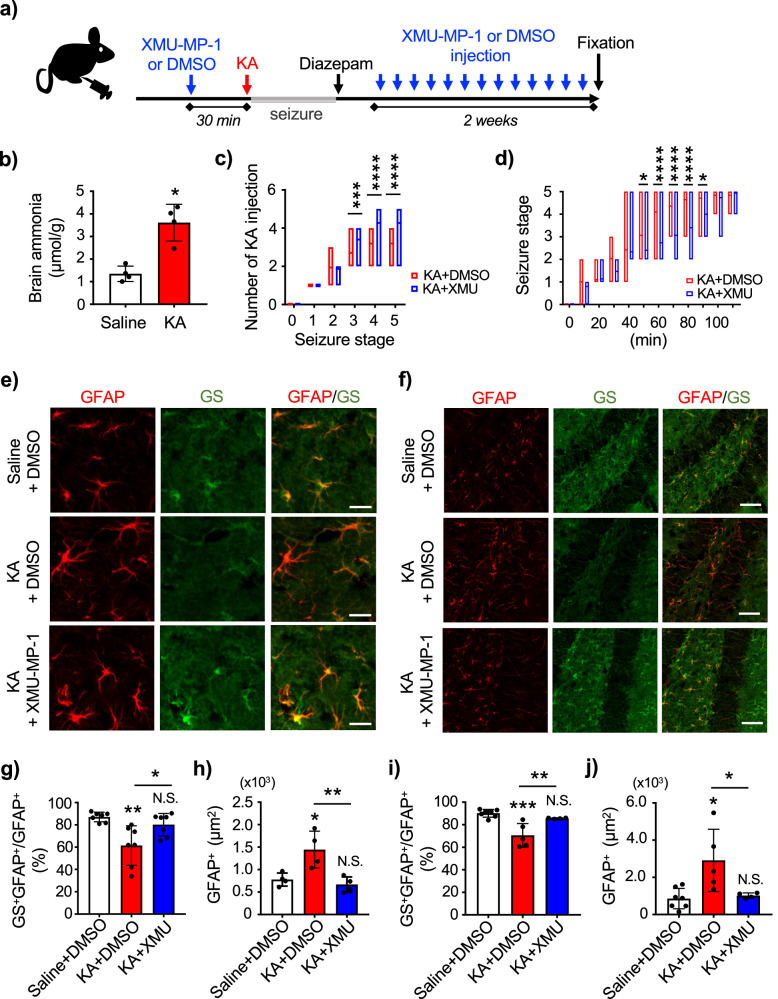
XMU-MP-1 recovers astrocytic GS expression in epileptic mouse brain. **a** Experimental design of generating kainic acid (KA)-induced status epilepticus mice. **b** Brain ammonia levels in the hippocampus of saline and KA-injected mouse. n = 4 animals per group. Mann-Whitney U test, *p < 0.05. **c** Number of KA injection at each seizure stage in mice pre-injected with DMSO (red) or XMU-MP-1 (blue). Floating bars show the minimum/maximum value and the mean. KA + DMSO n = 31 mice, KA + XMU-MP-1 n = 15 mice. One-way ANOVA followed by Sidak’s multiple comparisons test, ***p < 0.001, ****p < 0.0001. **d** Seizure progression by KA injection in mice pre-injected with DMSO (red) or XMU-MP-1 (blue). Mice received KA every 20 min until they reach stage 4 or 5 seizure. Floating bars show the minimum/maximum value and the mean. KA + DMSO n = 31 mice, KA + XMU-MP-1 n = 15 mice. One-way ANOVA followed by Sidak’s multiple comparisons test, *p < 0.05, ****p < 0.0001. **e** Visualization of hippocampal CA1 astrocytes in mice injected with Saline + DMSO, KA + DMSO, and KA + XMU-MP-1. Expression of GFAP (red) and GS (green) are shown. Scale bar 20 μm. **f** Visualization of hippocampal dentate gyrus astrocytes in mice injected with Saline + DMSO, KA + DMSO, and KA + XMU-MP-1. Expression of GFAP (red) and GS (green) are shown. Scale bar 50 μm. **g** Quantification of GS colocalization with GFAP in hippocampal CA1 in mice injected with Saline + DMSO, KA + DMSO, and KA + XMU-MP-1. n = 7 animals per group. One-way ANOVA followed by Tukey’s multiple comparisons test, *p < 0.05, **p < 0.01. N.S. = not significant. **h** Quantification of GFAP positive area in hippocampal CA1 in mice injected with Saline + DMSO, KA + DMSO, and KA + XMU-MP-1. n = 4 animals per group. One-way ANOVA followed by Tukey’s multiple comparisons test, *p < 0.05, **p < 0.01. N.S. = not significant. **i** Quantification of GS colocalization with GFAP in hippocampal dentate gyrus in mice injected with Saline + DMSO, KA + DMSO, and KA + XMU-MP-1. Saline + DMSO: n = 7, KA + DMSO: n = 5, KA + XMU-MP-1: n = 4 animals per group. One-way ANOVA followed by Tukey’s multiple comparisons test, **p < 0.01, ***p < 0.001. N.S. = not significant. **j** Quantification of GFAP positive area in hippocampal dentate gyrus in mice injected with Saline + DMSO, KA + DMSO, and KA + XMU-MP-1. Saline + DMSO: n = 7, KA + DMSO: n = 5, KA + XMU-MP-1: n = 4 animals per group. One-way ANOVA followed by Tukey’s multiple comparisons test, *p < 0.05. N.S. = not significant.

**Fig. 8:**
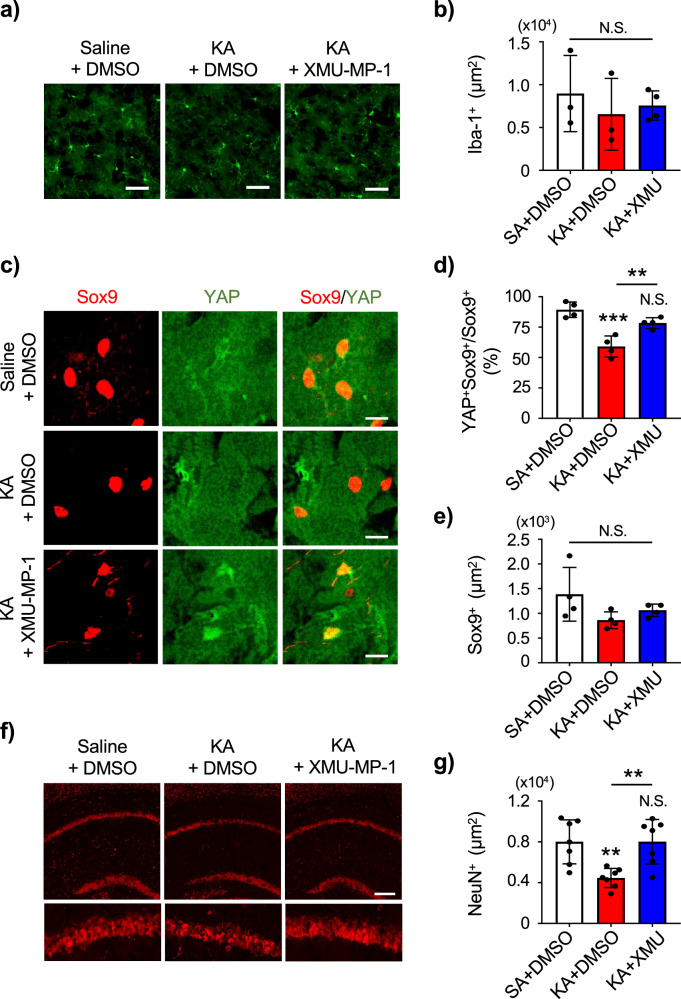
XMU-MP-1 induces YAP nuclear translocation and inhibits neuronal death in epileptic mouse brain. **a** Visualization of microglia in hippocampal CA1 in mice injected with Saline + DMSO, KA + DMSO, and KA + XMU-MP-1. Expression of Iba1 is shown. Scale bar 50 μm. **b** Quantification of Iba-1 positive area in hippocampal CA1 in mice injected with Saline (SA) + DMSO, KA + DMSO, and KA + XMU-MP-1. SA + DMSO: n = 3, KA + DMSO: n = 3, KA + XMU-MP-1: n = 4 animals per group. One-way ANOVA followed by Tukey’s multiple comparisons test. N.S. = not significant. **c** Visualization of hippocampal CA1 astrocytic nuclei and YAP in mice injected with SA + DMSO, KA + DMSO, and KA + XMU-MP-1. Expression of Sox9 (red) as a marker of astrocyte nucleus and YAP (green) are shown. Scale bar 10 μm. **d** Quantification of YAP colocalization with astrocyte nuclei marker Sox9. n = 4 animals per group. One-way ANOVA followed by Tukey’s multiple comparisons test, **p < 0.01, ***p < 0.001. N.S. = not significant. **e** Quantification of Sox9 positive area in hippocampal CA1 in mice injected with SA + DMSO, KA + DMSO, and KA + XMU-MP-1. n = 4 animals per group. One-way ANOVA followed by Tukey’s multiple comparisons test. N.S. = not significant. **f** Visualization of hippocampal CA1 neuron in mice injected with SA + DMSO, KA + DMSO, and KA + XMU-MP-1. Expression of NeuN (red) is shown. Scale bar 50 μm. **g** Quantification of NeuN positive area in hippocampal CA1 in mice injected with SA + DMSO, KA + DMSO, and KA + XMU-MP-1. n = 7 animals per group. One-way ANOVA followed by Tukey’s multiple comparisons test, **p < 0.01. N.S. = not significant.

To examine the effect of XMU-MP-1 in the symptom and pathology of epilepsy, we used a mouse model of status epilepticus (SE) (Fig. 7a). C57BL/6J male mice at 8- to 10-week-old age were intraperitoneally injected with saline or kainic acid (KA), an analog of glutamate. As expected, the concentration of ammonia was significantly elevated in the KA-injected epileptic mouse hippocampus (Fig. 7b). Continuous injection of KA at 20 min interval can develop SE in C57BL/6J mice (Fig. 7c), but the mice received XMU-MP-1 prior to KA injection (KA + XMU-MP-1 mice) required additional injection of KA to reach to the same seizure stage compared to that in DMSO-pretreated epileptic mice (KA + DMSO mice). Although KA + XMU-MP-1 mice showed significantly slower seizure progression than KA + DMSO mice, the mice eventually developed stage 5 seizure by additional injection of KA (Fig. 7d). The XMU-MP-1-pre-treated epileptic mice were further received XMU-MP-1 once a day for 2 weeks after the termination of seizures with diazepam, and the control group received DMSO (Fig. 7a). We found that following SE, astrocytes were hypertrophied, and the expression of GFAP was significantly increased in the hippocampal CA1 compared to Saline + DMSO mice (Fig. 7e, h). Accompanying with increased GFAP in the CA1 following SE, reduction of GS was observed in astrocytes (Fig. 7e, g). In contrast, the KA + XMU-MP-1 mice did not increase GFAP expressions in CA1 astrocytes and the expression level of GS was as same as Saline + DMSO mice (Fig. 7e, g, h). The administration of KA not only affected hippocampal CA1 but also hippocampal dentate gyrus (Fig. 7f ). The KA administration increased the number of reactive astrocytes and reduced the co-localization of GFAP and GS in the dentate gyrus, which were reversed by the XMU-MP-1 treatment (Fig. 7f, i, j). Since astrocyte reactivity and activated microglia are a hallmark of neuroinflammation, we then examined the activity of microglia in hippocampal CA1. No change was observed in Ionized calcium binding adaptor molecule 1 (Iba1), a marker for microglia in the CA1 (Fig. 8a, b).

Next, we examined whether the effect of XMU-MP-1 on GS expression in KA-injected mouse hippocampus were due to enhanced nuclear translocation of YAP in astrocytes. We found that the co-localization of YAP with Sox9 was decreased in the CA1 of KA + DMSO mouse hippocampus, and it was recovered by the XMU-MP-1 treatment (Fig. 8c, d). Of note, the seizure activity did not alter Sox9 expression (Fig. 8e). We also performed GS/YAP co-staining and confirmed that GS expression is in sync with YAP expression (Supplementary Fig. 6).

In our studies, XMU-MP-1 pre-treatment reduced seizure progression (Fig. 7d). Thus, we examined if pre-treatment of XMU-MP-1 is sufficient to block KA-induced neuroinflammation and GS reduction in astrocytes. We treated mice with XMU-MP-1 or DMSO 30 min prior to KA injection, developed seizure and terminated with diazepam. Mice were kept for 2 weeks following seizure termination without any injection and hippocampal CA1 was examined. We found that pre-treatment of XMU-MP-1 fail to block GS reduction induced by KA and did not suppress GFAP expression in astrocytes, that were elevated in KA + DMSO-injected mice (Supplementary Fig. 7a, c, d). We also found that reduced YAP expression in KA + DMSO-injected mouse astrocyte nucleus was not recovered by pre XMU-MP-1 treatment (Supplementary Fig. 7b, e). The Sox9 positive area was not different between the experimental mice (Supplementary Fig. 7f ). Together, these data suggested that only the XMU-MP-1 treatment after KA-induced seizure termination is effective to induce YAP nuclear localization and GS expression, which also inhibits neuroinflammation.

KA-induced seizure is known to cause neuronal loss in CA1 pyramidal neurons^28^. Thus, we observed the neuronal nuclei stained with NeuN in hippocampal CA1. In the Saline + DMSO-injected mice, NeuN immunoreactive ^(+)^ (NeuN + ) cells were observed in the CA1 pyramidal cell layer, while the immunoreactivity of NeuN+ cells in the KA + DMSO-injected epileptic mice were significantly reduced (Fig. 8f, g). However, seizure-induced loss of NeuN was suppressed in the KA + XMU-MP-1-treated epileptic mice (Fig. 8f, g). Supporting these observations, the high mortality rates found in the KA-administered group was diminished in XMU-MP-1-treated group (Table 1). Since the pre-treatment of XMU-MP-1 fail to confer its protective effect to CA1 neurons (Supplementary Fig. 8a, b), the recovery of GS expression in astrocytes by XMU-MP-1 treatment after seizure may have a neuroprotective role.

**Table 1:** Mortality rate of KA-injected mice with and without XMU-MP-1 administration

	KA + DMSO	KA + XMU-MP-1
Total number	38	16
Death during SE	7	1
Death after SE (24 h < )	7	0
Mortality rate during SE	18.42% (7/38)	6.25% (1/16)
Mortality rate after SE	22.58% (7/31)	0% (0/15)

Death during SE: animals died during stage 1–5 seizure. Death after SE: animals which received diazepam after SE but died before termination. The mortality rate of Saline + DMSO group after injection was 5.55% (1/18 animals).

**Fig. 9:**
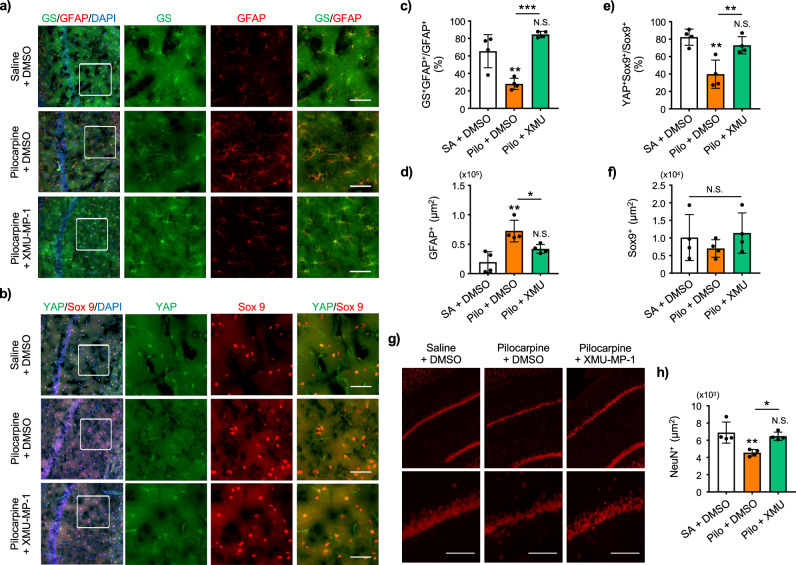
XMU-MP-1 induces YAP nuclear translocation, recovers astrocytic GS expression and inhibits neuronal death in pilocarpine-induced epileptic rat brain. **a** Visualization of hippocampal CA1 astrocytes in mice injected with Saline (SA) + DMSO, Pilocarpine + DMSO, and Pilocarpine + XMU-MP-1. Expression of GFAP (red) and GS (green) are shown. Nucleus was identified by DAPI (blue) staining. Scale bar 50 μm. **b** Visualization of hippocampal CA1 astrocytic nuclei and YAP in mice injected with SA + DMSO, Pilocarpine + DMSO, and Pilocarpine + XMU-MP-1. Expression of Sox9 (red), YAP (green) are shown. Nucleus was identified by DAPI (blue) staining. Scale bar 50 μm. **c** Quantification of GS colocalization with GFAP in hippocampal CA1 in mice injected with SA + DMSO, Pilocarpine + DMSO, and Pilocarpine + XMU-MP-1. **d** Quantification of GFAP positive area in hippocampal CA1. **e** Quantification of YAP colocalization with Sox9. **f** Quantification of Sox9 positive area in hippocampal CA1. **g** Visualization of hippocampal CA1 neuron in mice injected with SA + DMSO, Pilocarpine + DMSO, and Pilocarpine + XMU-MP-1. Expression of NeuN (red) is shown. Scale bar 50 μm. **h** Quantification of NeuN positive area in hippocampal CA1. SA + DMSO, Pilocarpine + DMSO, and Pilocarpine + XMU-MP-1. n = 4 animals per group. One-way ANOVA followed by Tukey’s multiple comparisons test, *p < 0.05, **p < 0.01, ***p < 0.001. N.S. = not significant.

Finally, to examine whether the observed astrocytic changes are consistent across different epilepsy models, we employed the pilocarpine-induced rat model, which is widely used as a model of temporal lobe epilepsy. Like KA-induced epilepsy mouse model, pilocarpine-treated rats exhibited increased GFAP expression in the hippocampal CA1 region, accompanied by a marked decrease in GS expression in GFAP-positive astrocytes (Fig. 9a, c, d). Post-treatment with XMU-MP-1 for 7 days restored both GFAP and GS expression levels to those comparable with Saline + DMSO-injected animals (Fig. 9a, c, d).

In addition, the nuclear translocation of YAP, assessed by its co-localization with Sox9, was significantly reduced in pilocarpine-treated rats. This reduction was not observed in the XMU-MP-1-treated group (Fig. 9b, e, f ). The NeuN+ cells were reduced in pilocarpine-treated rats, indicating neuronal loss or damage in the CA1 hippocampal neurons, and these reductions were attenuated in post- XMU-MP-1-injected rats (Fig. 9g, h).

## Discussion

GS in astrocytes plays an important role for maintaining the concentration of extracellular glutamate and ammonia in the brain. Deficiency of GS has been shown in various neurological disorders, but the molecular mechanism regulating the expression of GS and the mechanism of GS reduction in diseased brain is largely unknown. Here we identified that prolonged glutamate/NH_4_Cl treatment reduce GS and lower intracellular glutamine level. The reduction of GS was primarily induced by NH_4_Cl. However, the co-application of glutamate with NH_4_Cl reduced GS much more than NH_4_Cl alone. Since GS reduction in astrocytes has been reported from epileptic patients’ brain^29,30^ and is thought as a pathogenesis of epilepsy^31^, we wanted to identify how NH_4_Cl reduces GS in astrocytes.

Ammonia has been found to induce autophagy, and it is believed to be a mechanism for cells to protect from external stresses^17–19^. Furthermore, ammonia is a weak base that can cross cell membranes and neutralize acidic compartments. Thus, ammonia not only induces autophagy but also prevents lysosomal protease activity by raising intracellular pH. We determined a half-life of astrocytic GS with/without glutamate and NH_4_Cl administration, but we could not see differences between two treatments, suggesting that GS degradation is intact during glutamate/NH_4_Cl exposure. In addition, GS reduction was not blocked by the proteasomal inhibitor MG-132 or lysosome inhibitor leupeptin. These findings suggested that reduced GS expression by glutamate/NH_4_Cl co-treatment is not because of the degradation. Although, glutamate has been found to stimulate glutamate receptor interacting protein 1 degradation by ubiquitin-proteasome system^32^.

**Fig. 10:**
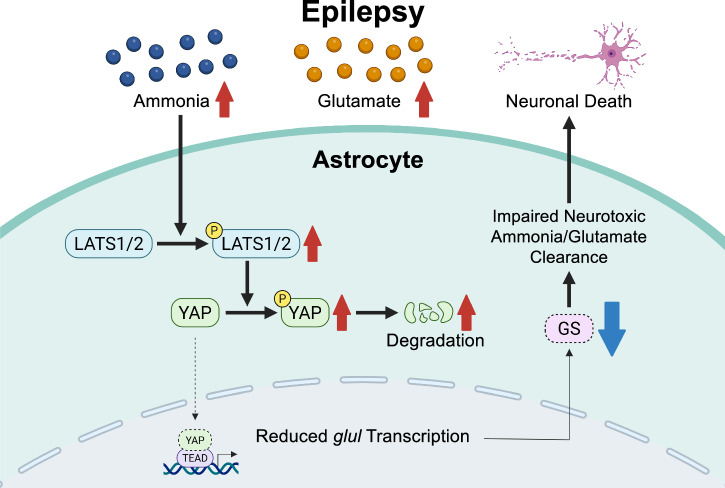
Schematic depicting how elevated ammonia and glutamate reduce GS expression in astrocytes. In the pathological condition when extracellular ammonia level is increased such as epilepsy, ammonia inhibits the nuclear localization of YAP through activation of Hippo pathway. Reduced YAP in the nucleus resulted in the reduction of GS that blocks metabolism of neurotoxic agent glutamate and ammonia. The pharmacological induction of YAP nuclear localization with XMU-MP-1 can recover GS expression and protects neurons against cell death.

The expression of GS is upregulated in cells that require continuous and rapid growth such as cancers^33,34^. It is found that hepatic GS enhances hepatocellular carcinoma development by activating mTOR complex 1 driven by β-catenin^35,36^. GS is also shown to induce hepatomegaly and promote tumorigenesis in zebrafish liver cancer model^9^. We found that YAP which is a regulator of cell proliferation modulates GS expression in astrocytes. YAP is known as a transcriptional coactivator, downstream effectors of the Hippo pathway^37^. When Hippo signaling pathway is active (Hippo ‘ON’), multiple upstream signals induce phosphorylation of YAP by the upstream MST1/2-LATS1/2, promotes the interaction of YAP with cytoskeletal proteins and stimulates retention of YAP in cytosol or proteolytic degradation^24^. In contrast, when Hippo pathway is inactive (Hippo ‘OFF’), dephosphorylated YAP enter the nucleus, binds with the transcription factor TEA domain family members (TEADs), and control the expression of target genes^38^. Currently known target genes of YAP-TEAD complex include *CTGF*, *CYR61*, *MYC* and *CCND1*, that are related to cell growth, proliferation and migration^39^. Our studies showed that astrocyte proliferation is inhibited by glutamate/NH_4_Cl. In addition, the inhibition of MST1/2 by XMU-MP-1 in the presence of glutamate and NH_4_Cl induced YAP nuclear translocation and upregulated GS expression, suggesting that astrocytes become Hippo ‘ON’ state by the glutamate/NH_4_Cl (Fig. 10). We also found that XMU-MP-1 treatment can enhance GS expression, suggesting that Hippo pathway regulates astrocytic YAP-GS axis in the physiological condition. In current study, we could only identify that NH_4_Cl activates LATS1 to suppress YAP. Further studies are needed to determine the upstream target of Hippo signaling. Furthermore, treatment of NH_4_Cl with glutamate reduced *glul* even more than a single NH_4_Cl treatment. Although glutamate stimulation did not alter astrocytic GS, G-protein-coupled receptor signaling has been shown to regulate Hippo-YAP pathway in several cell lines^40^. Thus, a cross talk between ammonia- and glutamate-mediated signaling may also be existed in astrocytes.

Since we identified that Hippo pathway kinase MST1/2 inhibitor XMU-MP-1 recovers GS in hyperammonemic condition, we prepared two rodent model of epilepsy and examined if XMU-MP-1 has an anti-epileptic effect by targeting astrocytic YAP and GS. To our surprise, pre-treatment of mice with XMU-MP-1 suppressed the development of seizures and mortality. The effect of XMU-MP-1 is remarkable and required an additional injection of kainic acid to reach stage 5 seizure. Because XMU-MP-1 was only administered 30 min prior to kainic acid injection, delayed seizure development is more likely not through induction of YAP nuclear localization. So far, XMU-MP-1 is only reported as an inhibitor of MST1/2. The pharmacological effect of XMU-MP-1 was first reported in the mouse model of acute and chronic liver injuries^41^. Recent studies in rodents shown that XMU-MP-1 can reduce myocardial ischemia/reperfusion injury and ischemia/stroke-induced brain injury as well^42,43^. Furthermore, pre-treatment of mice with XMU-MP-1 markedly alleviated the injury at the small intestinal system caused by the body irradiation and extended the survival days of mice exposed with the lethal dose of radiation^44^. We found that XMU-MP-1 treatment recovers nuclear YAP expression and increases GS expression in astrocytes, which was significantly reduced in our epileptic rodents. XMU-MP-1 treatment also reduced neuronal loss observed in epileptic animals. However, pre-treatment of XMU-MP-1 did not have effect on GS expression. These results suggested that temporarily turning off the Hippo pathway during seizure may be an effective treatment for epilepsy. Together, our work identifies that YAP may be an effective target for diseases in which astrocytic GS expression is reduced due to elevated ammonia.

## Materials and methods

### Animals

To prepare cerebral cortical astrocyte cultures, E18 to 19 Sprague-Dawley rats (SD/Jcl, CLEA Japan) were used. To generate the mouse model of status epilepticus, 8-10-week-old male C57BL6/J mice (Jackson Laboratory) were used. To generate YAP conditional knockout mice in which YAP is selectively knockout in astrocytes, the YAP^flox/flox^ mice^25^ and Aldh1l1-CreER^T2^ mice^26^ were crossed. All experiments were carried out in accordance with the Guidelines for the Care and Use of Laboratory Animals of Niigata University. Animal care and experimental protocols were approved by the Animal Experiment Committee of the Niigata University (approval No. SA01161, SA01166, SA01249, SA01585, and SA01586).

### Preparation of primary astrocyte cultures

Cerebral cortical and hippocampal astrocytes were prepared from E18 to 19 Sprague-Dawley rats as previously described^45^. For the cellular treatments, following chemicals were used: NH_4_Cl (Sigma-Aldrich), glutamate (FUJIFILM Wako), 0.2 μM MG-132 (Chem Scene)^46^, 5 μM cycloheximide (Sigma-Aldrich)^45^, Leupeptin (Sigma-Aldrich)^47^, 1 μM Verteporfin (Sigma-Aldrich)^48^, 20 µM FH535 (Cayman)^49^, 3 μM XMU-MP-1 (Cayman)^50^, 40 μM SKL2001 (Cayman)^51^, DMSO (FUJIFILM Wako). Both glutamate and NH_4_Cl were dissolved in ultrapure water, and all the other reagents were dissolved in DMSO. Final concentration of DMSO in culture media was below 0.1%.

### Western blotting

Standard western blot protocol was used as described previously^47^. Protein samples were subjected to SDS-PAGE and transferred to supported nitrocellulose membranes (Cytiva). Membranes were stained with ponceau S (Sigma-Aldrich) for protein detection, then blocked with blocking buffer (5% bovine serum albumin (BSA) in Tris Buffered Saline with Tween 20). Proteins of interest were probed with primary antibodies against GS (Millipore, MAB302), YAP (Cell Signaling Technology, D8H1X), phospho-YAP (Ser127, Cell Signaling Technology, D9W2I), α-tubulin (Sigma-Aldrich, T5168), GAPDH (Santa Cruz Biotechnology, SC365062), Lamin B1 (Santa Cruz Biotechnology, SC377000), LATS1 (Cell Signaling Technology, C66B5), phospho-LATS1 (Ser909, Cell Signaling Technology, 9157), MST1 (Cell Signaling Technology, 3682), phospho-MST1/2 (Cell Signaling Technology, E7U1D), then probed with horseradish peroxidase–conjugated secondary antibodies (GE Healthcare) and visualized by ECL (SuperSignal West Dura Extended Duration Substrate, Thermo Fisher Scientific). Blots were quantified using the CCD-based Amersham Imager 680 system (GE Healthcare Life Sciences) and the intensity of bands was measured using Image J.

### Immunocytochemistry

Astrocytes on coverslips were fixed in 4% paraformaldehyde (FUJIFILM Wako) for 15 min at room temperature. Fixed cells were permeabilized in 0.2% Triton X-100 (Sigma-Aldrich) or 0.01% Saponin (Sigma-Aldrich) for 30 min at room temperature. Following blocking in PBS supplemented with 0.2% Triton X-100 and 1% BSA (FUJIFILM Wako), cells were incubated with the following primary antibodies overnight at 4 °C: anti-GS (Millipore, MAB302), anti-YAP (Cell Signaling Technology, D8H1X). After washing with PBS, cells were then incubated with fluorescently tagged secondary antibodies (VECTOR Laboratories) for 1 h at room temperature. All cells were counterstained with DAPI by using mounting medium containing DAPI (VECTOR Laboratories). Confocal images were taken using a laser-scanning confocal microscope (Zeiss LSM700) and images were analyzed using Image J.

### Cell counting

Astrocytes grown in 6 cm dishes were harvested by applying trypsin-EDTA (Gibco) and the collected cells were centrifuged at 1,000 rpm for 5 min. Cell pellets were then resuspended in 1 ml of Hank’s balanced salt solution (Gibco) and a portion of cell suspension was counted using a TC20 automated cell counter (Bio-Rad Laboratories).

### RT-PCR

Total RNA from cultured astrocytes was extracted using an RNeasy mini kit (Qiagen). RNA was quantified using a NanoDrop One (Thermo Fisher Scientific) and then retro-transcribed using oligo (dT)_20_ Primer (Thermo Fisher Scientific), M-MLV Reverse Transcriptase (Promega), Recombinant ribonuclease inhibitor (Invitrogen), and dNTPs (TOYOBO). The cDNA was subjected to PCR with Taq (Takara), dNTPs (TOYOBO), and following primers: *GS (GLUL)*, 5’-CGAAGTATTCAAGTATAACCGGAAG-3’ and 5’-TCACCATGTCCATTATAC GCTTAC-3’; *GAPDH*, 5′-GGCAAGTTCAATGGCACAGT-3′ and 5′CTCAGATGACCGCAGAAGTGGT-3’. PCR products were separated by electrophoresis on an agarose gel and stained with GelRed Nucleic Acid Stain (Biotium) for visualization. The intensity of bands was measured using Image J. The expression level of GS mRNA was normalized to the level of GAPDH.

For real-time PCR, the specific primers and probes for GS and GAPDH were purchased from Applied Biosystems. Real-time PCR was performed by the Step One Plus real time PCR system (Applied Biosystems) using TaqMan Fast Advanced Master Mix (Applied Biosystems). Analysis was performed by using the Step One Plus real time PCR software (Applied Biosystems) and the data were normalized to GAPDH.

### Preparation of cytosolic and nuclear fractionation in cultured astrocytes

Subcellular fractionation was conducted using the NE-PER Nuclear and Cytoplasmic Extraction Reagents kit (Thermo Fisher Scientific, 78833) according to the manufacturer’s protocol.

### Transfection of primary astrocytes

Transfection of primary astrocytes was performed using the Nucleofector transfection reagent and the electroporator according to the manufacturer’s protocol (Lonza, VPI-1006). Briefly, astrocytes at the passage 1 or 2 were trypsinized, and 4.0-6.0 × 10^6^ cells were subsequently applied to electroporation with 4.5 μg of reporter plasmid DNA and 2.0 μg of pmaxGFP vector. Cell suspension was gently transferred and seeded at 1.0 × 10^6^ cells in 6 cm culture dish. Cells were collected 48 h later when GFP expression was observed. The pEF-YAP-N-FLAG vector was a kind gift from Dr. Shimizu (Nagoya city university, Nagoya, Japan)^52^.

### Mouse model of status epilepticus

Kainic acid-induced status epilepticus (SE) mice were prepared by injecting kainic acid (5 mg/kg body weight, Sigma-Aldrich) intraperitoneally every 20 min until the first Racine stage 4/5 seizure was observed^53^. Epileptic behavior in mice was scored as the following stages: Stage Ⅰ: mouth and facial movements, Stage Ⅱ: head nodding, Stage Ⅲ: forelimb clonus, Stage Ⅳ: rearing, Stage Ⅴ: rearing and falling. Control mice were injected with saline. In some experiments, XMU-MP-1 (1 mg/kg body weight, Cayman)^41–43^ was intraperitoneally injected 30 min before kainic acid injection, then continuously injected once a day from the next day until day 14. XMU-MP-1 was dissolved in DMSO (stock concentration, 3 mM) and diluted in saline just before injection. Solution of 10% DMSO (in saline) was used as a negative control. Numbers of kainic acid injection in each seizure stages and chronological seizure progression were also evaluated. Diazepam (5 mg/kg body weight, Sigma-Aldrich) was intraperitoneally administered 2 h post SE. Mortality rate in each group is shown in Table 1. Fourteen days following SE, the surviving mice were anesthetized with sevoflurane (Maruishi Pharmaceutical Company) and tissues were harvested for the analysis. Brain ammonia levels were measured using an Ammonia Assay Kit (abcam, ab83360) and the content of glutamine and glutamate was measured using the Glutamine/Glutamate-Glo^TM^ Assay (Promega, J8021) according to the manufacturer’s instructions.

### Rat model of status epilepticus

Status epilepticus was induced in male Sprague-Dawley rats (7–10 weeks old) using a lithium–pilocarpine protocol^54,55^. Twenty-four hours prior to seizure induction, lithium chloride (127 mg/kg body weight, Sigma-Aldrich) was intraperitoneally administered. On the following day, scopolamine methyl bromide (1 mg/kg body weight, Sigma-Aldrich) was intraperitoneally injected to minimize peripheral cholinergic effects, then 30 min later, pilocarpine hydrochloride (10 mg/kg body weight, Sigma-Aldrich) was administered every 30 min until the animals reached Racine stage 4 or 5 seizures. Diazepam (10 mg/kg body weight, Sigma-Aldrich) was intraperitoneally administered 2 h post SE to terminate seizures. In the following day, rats received intraperitoneal injections of either XMU-MP-1 (0.5 mg/kg body weight, Cayman Chemical) or DMSO (FUJIFILM Wako) dissolved in saline once a day for 7 consecutive days.

### Immunohistochemistry

Under deep inhalation of sevoflurane (Maruishi Pharmaceutical Company), rodents were transcardially perfused with 4% paraformaldehyde in 0.1 M phosphate buffer (pH 7.2). Cryosections were prepared at 15 μm using a cryostat (Leica). For immunostaining, sections were permeabilized with 0.3% Triton X-100 in PBS for 15 min and then blocked with 0.5% skim milk (Megmilk Snow Brand) for another 15 min at room temperature. The sections were incubated with the following primary antibodies in 0.1% Triton X-100 containing PBS for overnight at 4 °C: monoclonal mouse anti-GFAP (Millipore, MAB360), polyclonal rabbit anti-GFAP (Millipore, AB5804), anti-Iba1 (FUJIFILM Wako, 019-19741), anti-NeuN (Millipore, MAB377), anti-YAP (Santa Cruz Biotechnology, sc101199), anti-YAP (Sigma-Aldrich, WH0010413M1), anti-GS (Millipore, MAB302), and anti-Sox9 (Millipore, AB5535). Sections were then incubated with Alexa Fluor 488- and/or Alexa Fluor 594-labeled species-specific secondary antibodies (Thermo Fisher Scientific) for 2 h. Images were taken with a confocal laser-scanning microscope (Zeiss LSM710; Carl Zeiss) or all-in-one fluorescence microscope (BZ-X800, KEYENCE) and images were analyzed using Image J. To visualize YAP, sections were blocked with 10% BSA for 1 h at room temperature after permeabilization. To visualize GS, antigen retrieval was performed with 10 mM Citate Buffer (pH 6.0) for 30 sec at 95 °C. The sections were permeabilized with 0.5% Triton X-100 in PBS for 20 min and then blocked with 2% BSA for 1 h at room temperature.

### Statistical analysis

Data were subjected to paired *t* test, unpaired *t* test, Mann-Whitney U test, one-way or two-way ANOVA followed by Dunnett’s multiple comparisons test (one-way), Tukey’s multiple comparisons test (one-way) or Sidak’s multiple comparison tests (two-way), as appropriate with *p* < 0.05 as statistically significant. All statistical analysis were performed using GraphPad Prism 7.0 software. Values on the graph represent the mean ± SD. Experiments were repeated at least three times using different batches of cultures or animals.

### Reporting summary

Further information on research design is available in the Nature Portfolio Reporting Summary linked to this article.

## Supplementary information

Supplementary Information

Description of Additional Supplementary File

Supplementary Data 1

Reporting summary

## Data Availability

All uncropped images of western blot analysis are available in Supplementary Fig. 9. The source data supporting the findings of this study are available in Supplementary Data 1. Summary diagram (Fig. 10) was created using BioRender. All other data are available from the corresponding author on reasonable request.
