# Supplementary material for: Ammonia reduces glutamine synthetase expression in astrocytes via activation of Hippo-YAP signaling pathways
Source: Commun Biol. 2025 Dec 13;8:1810. doi: 10.1038/s42003-025-09191-5 (PMC12728173; doi:10.1038/s42003-025-09191-5)
Supplement: Supplementary file 1 — Supplementary Information [file 42003_2025_9191_MOESM1_ESM.pdf]

Supplementary Information

Ammonia reduces glutamine synthetase expression in astrocytes via activation of Hippo-YAP signaling pathways

Yusuke Nasu, Sari Kishikawa, Mamiko Imai, Nozomi Yokoyama, Izumi Iida, Koichi Tabeta, Miho Terunuma

Supplementary Figure 1

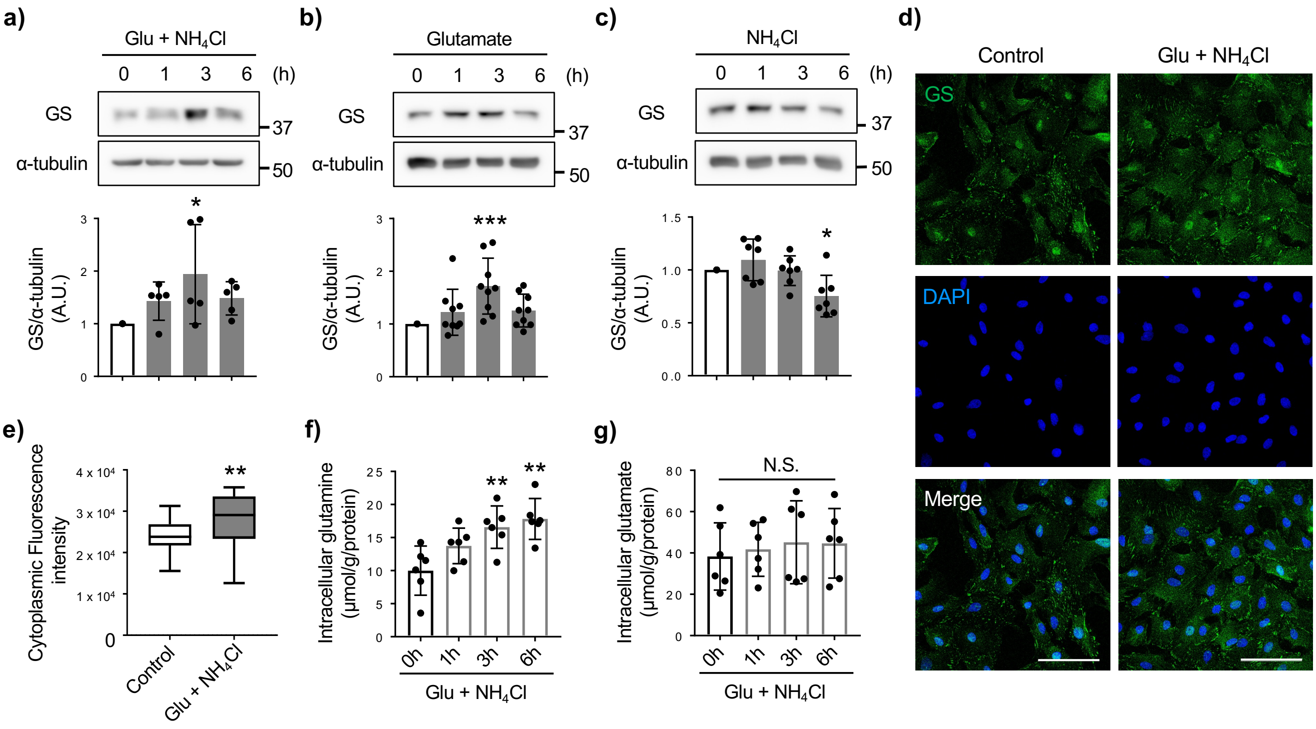

Supplementary Figure. 1 Glutamate elevates the expression of GS in cultured astrocytes

a Representative western blots of GS and α-tubulin after 20 µM glutamate (Glu) and 10 mM NH_4Cl co-treatment (Glu + NH_4Cl) in cultured astrocytes. Graph shows quantification of GS normalized to α-tubulin for each experiment and expressed as percent change versus 0 h condition. n = 5. One-way ANOVA followed by Dunnett's multiple comparisons test, *$p < 0.05$. **b** Representative western blots of GS and α-tubulin after 20 µM glutamate treatment in cultured astrocytes. Graph shows quantification of GS. n = 9. One-way ANOVA followed by Dunnett's multiple comparisons test, ***$p < 0.001$. **c** Representative western blots of GS and α-tubulin after 10 mM NH_4Cl treatment in cultured astrocytes. Quantification of GS. n = 7. One-way ANOVA followed by Dunnett's multiple comparisons test, *$p < 0.05$. **d** Representative images of immunofluorescence staining for GS (green) in cultured astrocytes with/without glutamate + NH_4Cl treatment for 3 h. Nucleus was identified by DAPI (blue) staining. n = 3. Scale bar 100 µm. **e** Quantification of cytoplasmic GS immunostaining in astrocytes with/without glutamate + NH_4Cl treatment for 3 h. Boxplot shows the minimum/maximum value and the median. Control: n = 30 cells, Glu + NH_4Cl: n = 30 cells. Mann-Whitney U test, **$p < 0.01$. **f, g** Measurement of intracellular glutamine (**f**) and glutamate (**g**) after glutamate + NH_4Cl treatment in cultured astrocytes. n = 6. One-way ANOVA followed by Dunnett's multiple comparisons test, **$p < 0.01$. N.S. = not significant.

Supplementary Figure 2

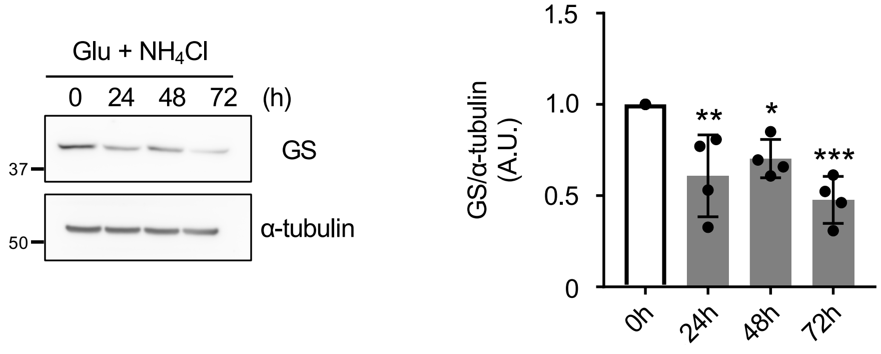

Supplementary Figure. 2 Prolonged glutamate/NH_4Cl treatments reduce the expression of GS in rat primary cultured hippocampal astrocytes

Left Representative western blots of GS and α-tubulin after 20 μM glutamate and 10 mM NH_4Cl co-treatments (Glu + NH_4Cl) for 0-72 h in rat primary cultured hippocampal astrocytes. **Right** Graph shows quantification of GS normalized to α-tubulin for each experiment and expressed as percent change versus 0 h condition. n = 4. One-way ANOVA followed by Dunnett's multiple comparisons test, *$p < 0.05$, **$p < 0.01$, ***$p < 0.001$.

Supplementary Figure 3

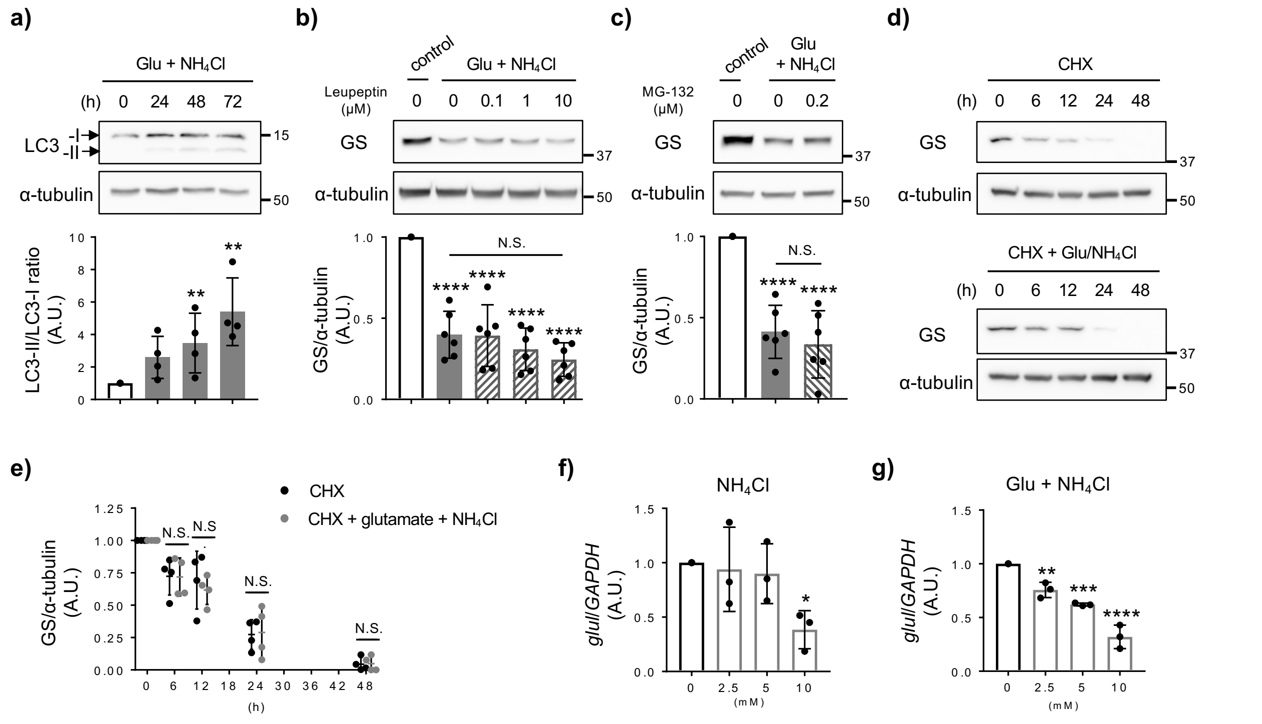

Supplementary Figure. 3 Glutamate and NH_4Cl co-treatments do not induce GS degradation but may alter protein synthesis

a Representative western blots of LC3-I, LC3-II and α-tubulin after 20 µM glutamate and 10 mM NH_4Cl (Glu + NH_4Cl) co-treatment in cultured astrocytes. Graph shows the ratio of LC3-II and LC3-I. Expression of both LC3-II and LC3-I were normalized using α-tubulin then the ratio of LC3-II/LC3-I were analyzed. n = 4. One-way ANOVA followed by Dunnett's multiple comparisons test, $^{**}p < 0.01$. **b** Representative western blots of GS and α-tubulin at various dose of leupeptin treatments for 72 h with 20 µM glutamate and 10 mM NH_4Cl co-treatment in cultured astrocytes. Graph shows quantification of GS normalized to α-tubulin for each experiment and expressed as percent change versus control condition. n = 6. One-way ANOVA followed by Tukey's multiple comparisons test, $^{****}p < 0.0001$, N.S = not significant. **c** Representative western blots of GS and α-tubulin after glutamate/NH_4Cl co-treatment together with/without MG-132 (0.2 µM) in cultured astrocytes. Graph shows quantification of GS. n = 6. One-way ANOVA followed by Tukey's multiple comparisons test, $^{****}p < 0.0001$, N.S = not significant. **d** Representative western blots showing the turnover of GS with (lower) or without (upper) 20 µM glutamate and 10 mM NH_4Cl co-treatment. Protein synthesis was blocked by 5 µM cycloheximide (CHX). **e** Quantification of GS expression in astrocytes that are blocked with CHX and treated with (gray circle) and without (black circle) 20 µM glutamate and 10 mM NH_4Cl. GS was normalized against α-tubulin. n = 4. Two-way ANOVA followed by Sidak's multiple comparisons test.

N.S = not significant. **f** Quantification of GS mRNA (*glul*) levels at various dose of NH_4Cl treatments for 72 h in cultured astrocytes by Real-Time PCR. Expression of *glul* was normalized to *GAPDH*. n = 3. One-way ANOVA followed by Dunnett's multiple comparisons test, $^{*}p < 0.05$. N.S. = not significant. **g** Quantification of *glul* at various dose of NH_4Cl treatments with 20 μM glutamate for 72 h in cultured astrocytes by Real Time PCR. Expression of *glul* was normalized to *GAPDH*. n = 3. One-way ANOVA followed by Dunnett's multiple comparisons test, $^{**}p < 0.01$, $^{***}p < 0.005$, $^{****}p < 0.0001$.

Supplementary Figure 4

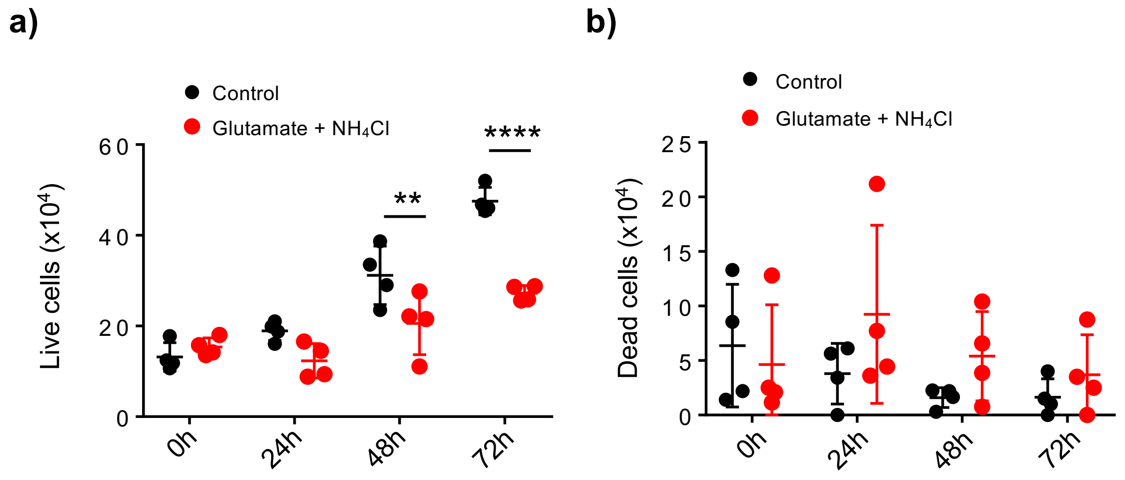

Supplementary Figure. 4 Glutamate and NH_4Cl co-treatment reduces proliferation of cultured astrocytes

a, b Cell count of the live (**a**) and dead (**b**) cells in astrocyte culture treated with glutamate and NH_4Cl. n = 4. Two-way ANOVA followed by Sidak's multiple comparisons test, $^{**}p < 0.01$, $^{****}p < 0.0001$.

Supplementary Figure 5

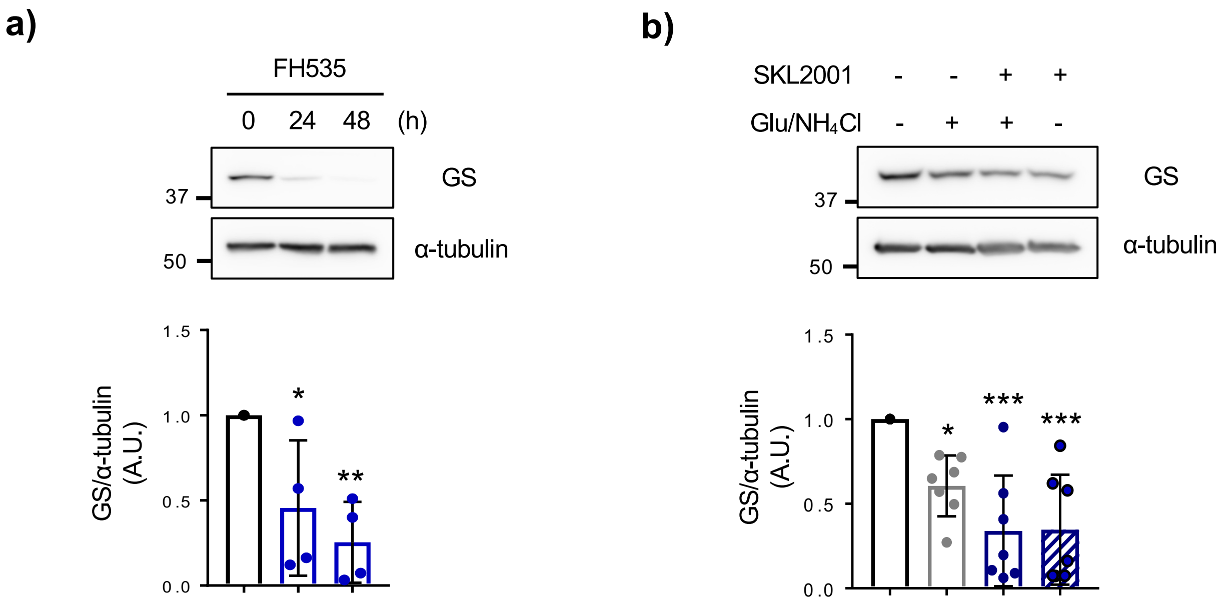

Supplementary Figure. 5 Wnt/β-catenin pathways act on GS expression but do not recover YAP expression reduced by glutamate/NH_4Cl co-treatments

a Representative western blots of GS and α-tubulin in cultured astrocytes treated with 20 µM FH535. Graph shows quantification of GS for each experiment and expressed as percent change versus 0 h condition. n = 4. One-way ANOVA followed by Dunnett's multiple comparisons test, *$p < 0.05$, **$p < 0.01$. **b** Representative western blots of GS and α-tubulin in astrocytes treated with glutamate + NH_4Cl and/or SKL2001 (40 µM) for 48 h. Graph shows quantification of GS for each experiment and expressed as percent change versus SKL2001(-)/Glu+NH4Cl(-) condition. n = 7. One-way ANOVA followed by Dunnett's multiple comparisons test, *$p < 0.05$, ***$p < 0.001$.

Supplementary Figure 6

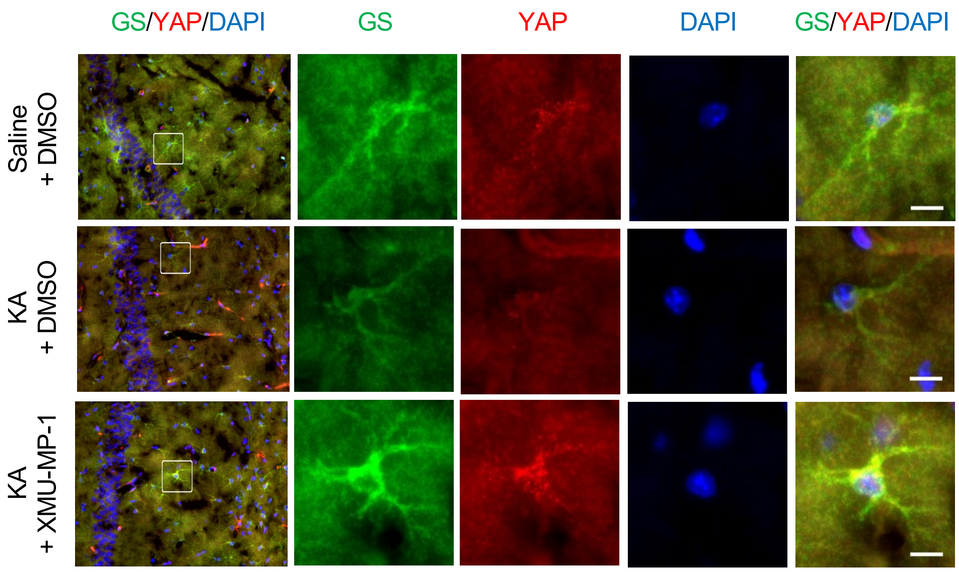

Supplementary Figure. 6 XMU-MP-1 recovers both GS and YAP expression in epileptic mouse brain
Visualization of hippocampal CA1 astrocytes in mice injected with Saline + DMSO, KA + DMSO, and KA + XMU-MP-1. Expression of GS (green) and YAP (red) are shown. Nucleus was identified by DAPI (blue) staining. Scale bar 10 µm.

Supplementary Figure 7

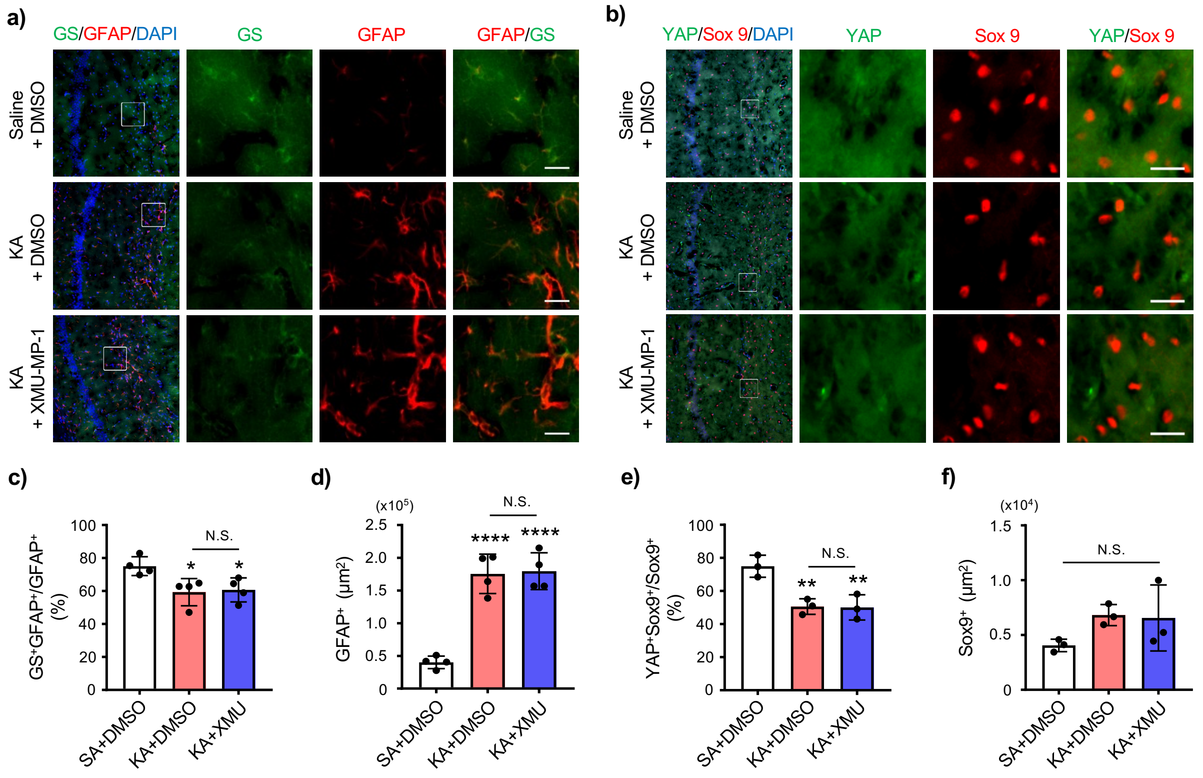

Supplementary Figure. 7 Pre-treatment of XMU-MP-1 does not recover astrocytic GS expression in epileptic mouse brain

a Visualization of hippocampal CA1 astrocytes in mice injected with Saline + DMSO, KA + DMSO, and KA + XMU-MP-1. Expression of GFAP (red) and GS (green) are shown. Nucleus was identified by DAPI (blue) staining. Scale bar 20 μm. **b** Visualization of hippocampal CA1 astrocytic nuclei and YAP in mice injected with SA + DMSO, KA + DMSO, and KA + XMU-MP-1. Expression of Sox9 (red) as a marker of astrocyte nucleus and YAP (green) are shown. Nucleus was identified by DAPI (blue) staining. Scale bar 20 μm. **c** Quantification of GS colocalization with GFAP in hippocampal CA1 in mice injected with Saline + DMSO, KA + DMSO, and KA + XMU-MP-1. n = 4 animals per group. One-way ANOVA followed by Tukey's multiple comparisons test, $*p < 0.05$. N.S. = not significant. **d** Quantification of GFAP positive area in hippocampal CA1 in mice injected with Saline + DMSO, KA + DMSO, and KA + XMU-MP-1. n = 4 animals per group. One-way ANOVA followed by Tukey's multiple comparisons test, $****p < 0.0001$. N.S. = not significant. **e** Quantification of YAP colocalization with astrocyte nuclei marker Sox9. n = 3 animals per group. One-way ANOVA followed by Tukey's multiple comparisons test, $**p < 0.01$. N.S. = not significant. **f** Quantification of Sox9 positive area in hippocampal CA1 in mice injected with SA + DMSO, KA + DMSO, and KA + XMU-MP-1. n = 3 animals per group. One-way ANOVA followed by Tukey's multiple comparisons test. N.S. = not significant.

Supplementary Figure 8

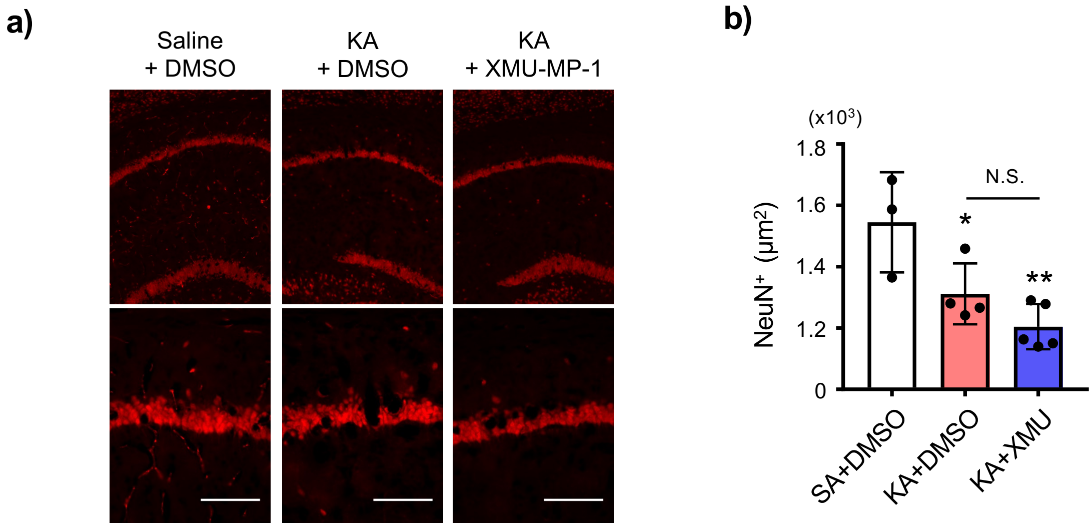

Supplementary Figure. 8 Pre-treatment of XMU-MP-1 does not inhibit neuronal death in epileptic mouse brain

a Visualization of hippocampal CA1 neuron in mice injected with SA + DMSO, KA + DMSO, and KA + XMU-MP-1. Expression of NeuN (red) is shown. Scale bar 100 µm. **b** Quantification of NeuN positive area in the hippocampal CA1 in mice injected with SA + DMSO, KA + DMSO, and KA + XMU-MP-1. SA + DMSO n = 3, KA + DMSO, n = 4, and KA + XMU-MP-1 n = 5 animals per group. One-way ANOVA followed by Tukey's multiple comparisons test, $*p < 0.05$, $**p < 0.01$. N.S. = not significant.

Supplementary Figure 9

Figure 1   b) and c) Images were taken using LAS3000, which cannot merge marker photo and chemiluminescent images

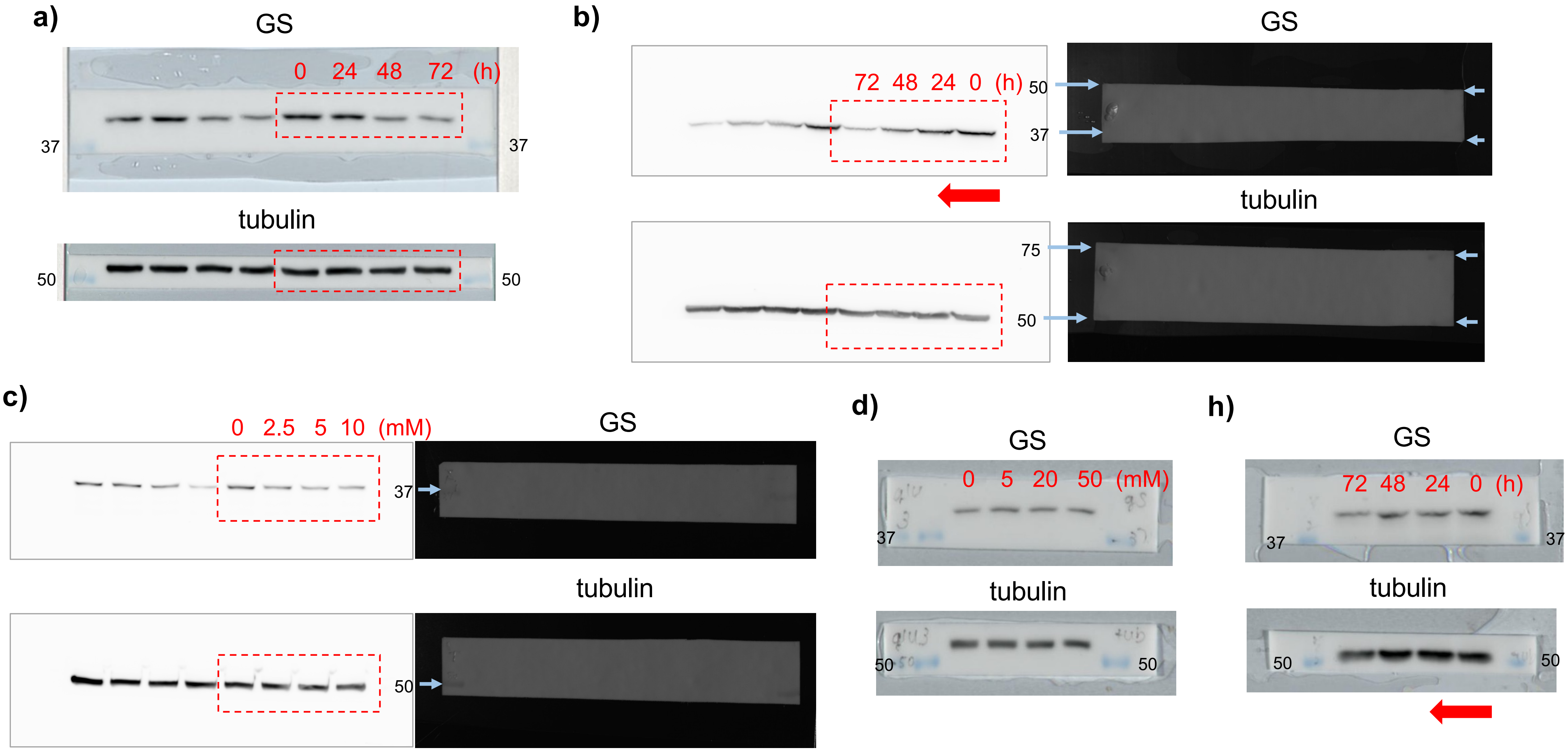

Figure 2

a)

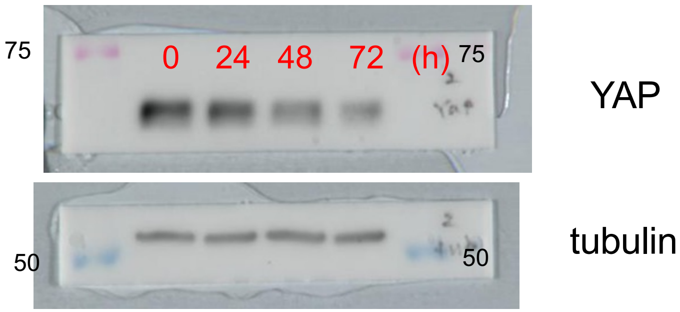

c)

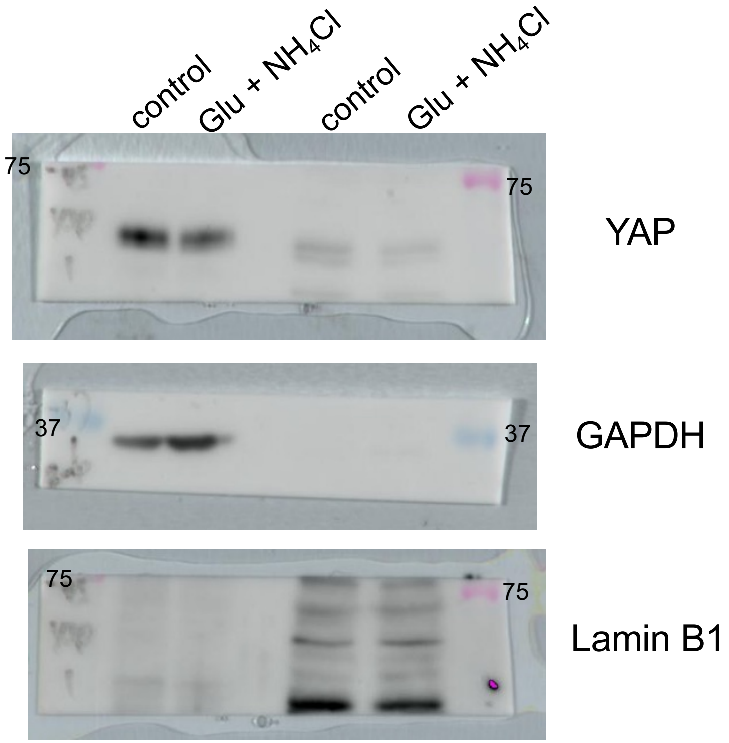

Membrane used to detect YAP was stripped and re-probed for Lamin B1

Figure 3

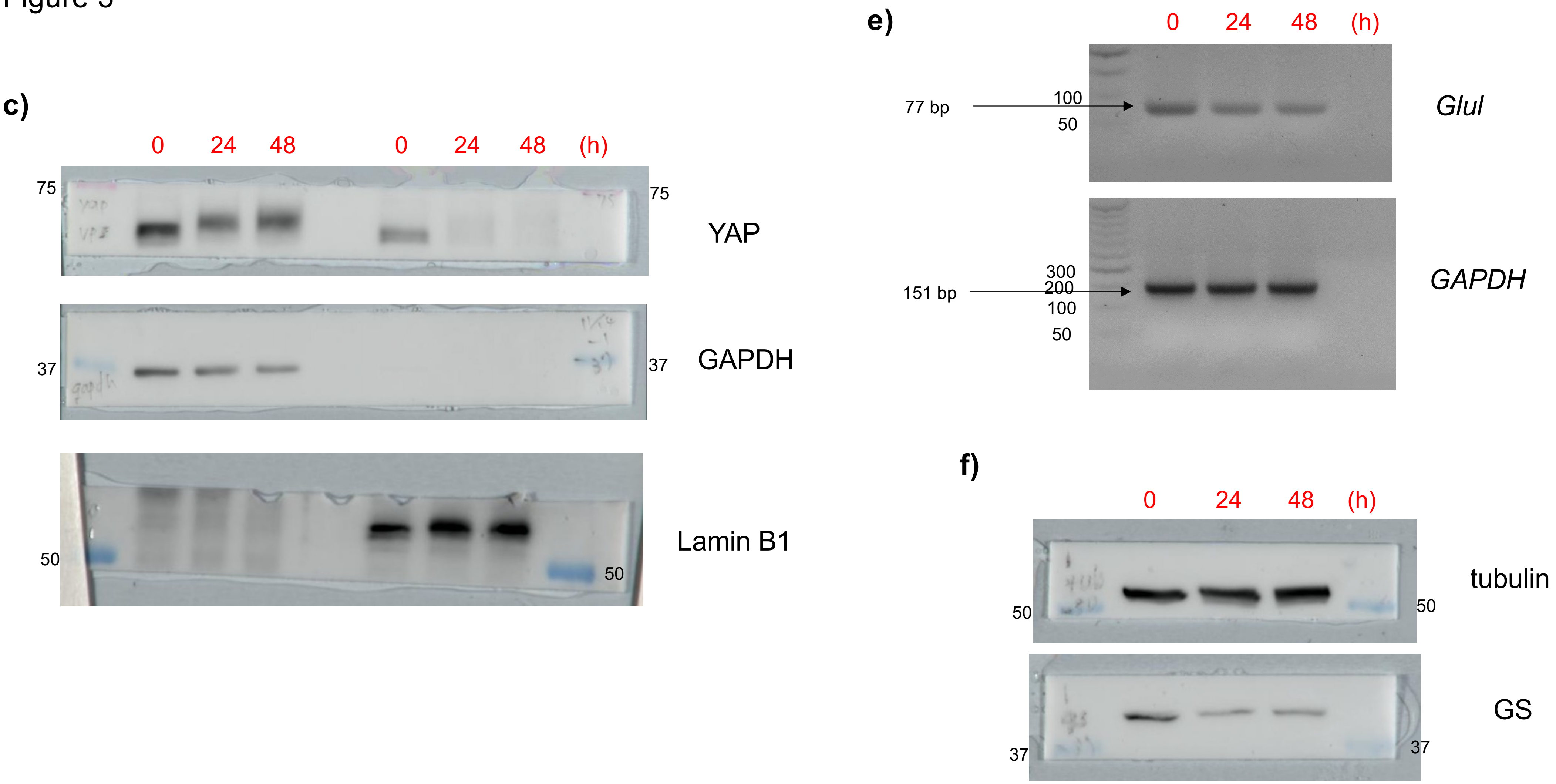
c)
0
24
48
0
24
48
(h)
75
75
YAP
37
37
GAPDH
50
50
Lamin B1
e)
0
24
48
(h)
77 bp
100
50
Glul
300
200
151 bp
100
50
GAPDH
f)
0
24
48
(h)
tubulin
50
50
GS
37
37

Figure 4

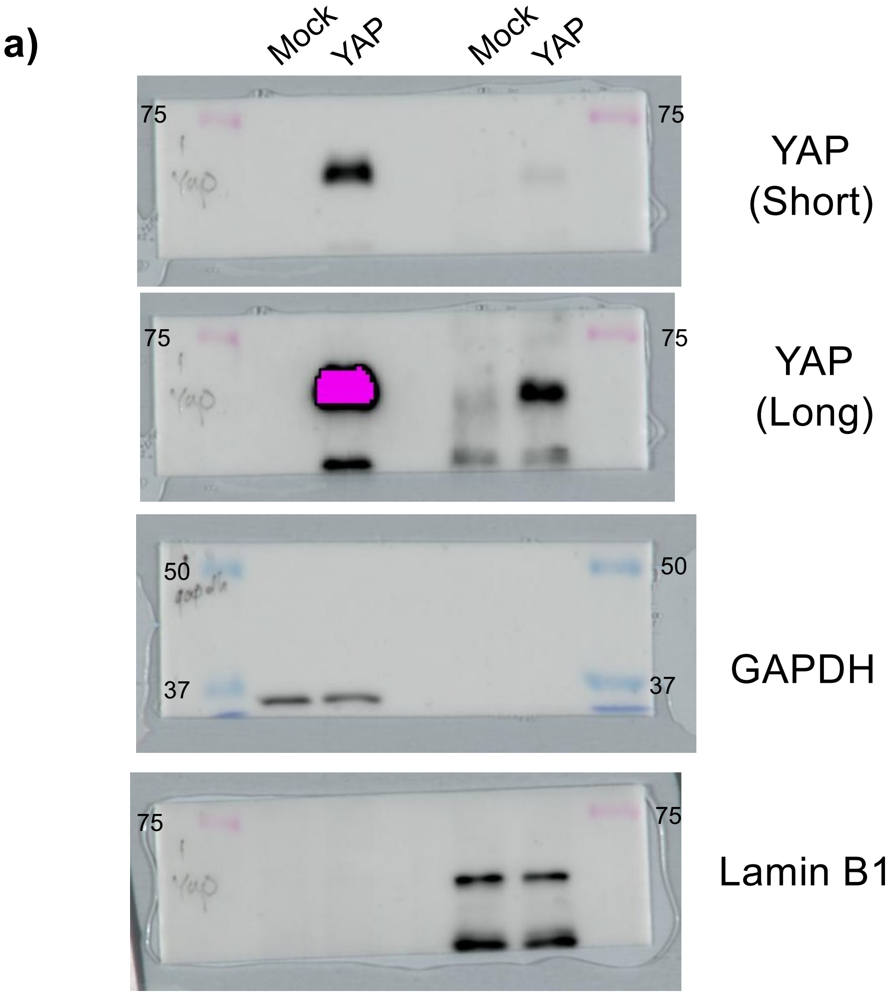
a)
Mock
YAP
Mock
YAP
75
75
YAP
(Short)
75
75
YAP
(Long)
50
50
37
37
GAPDH
75
75
Lamin B1

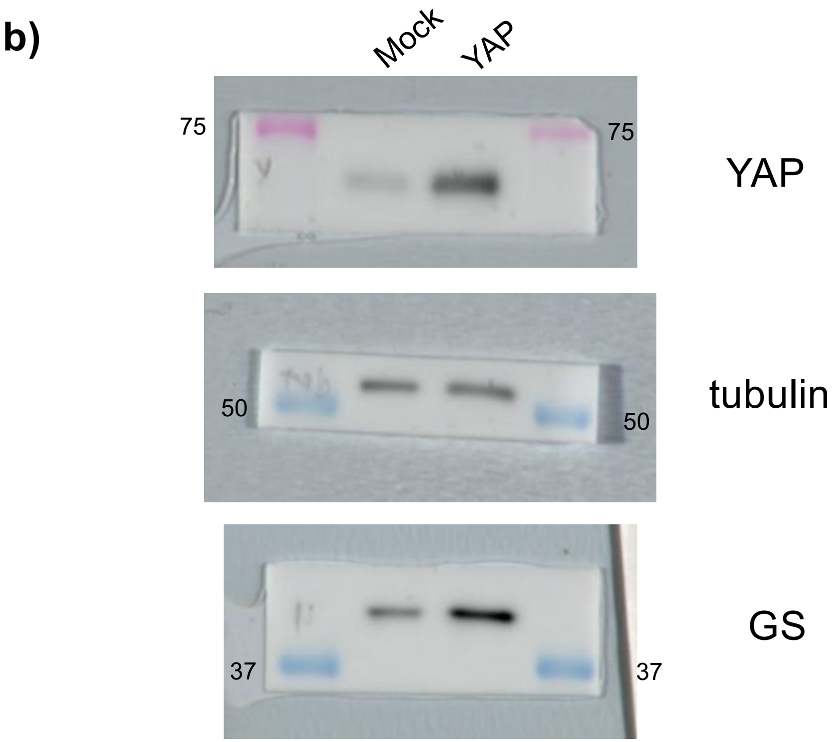
b)
Mock
YAP
75
75
YAP
50
50
tubulin
37
37
GS

Figure 5

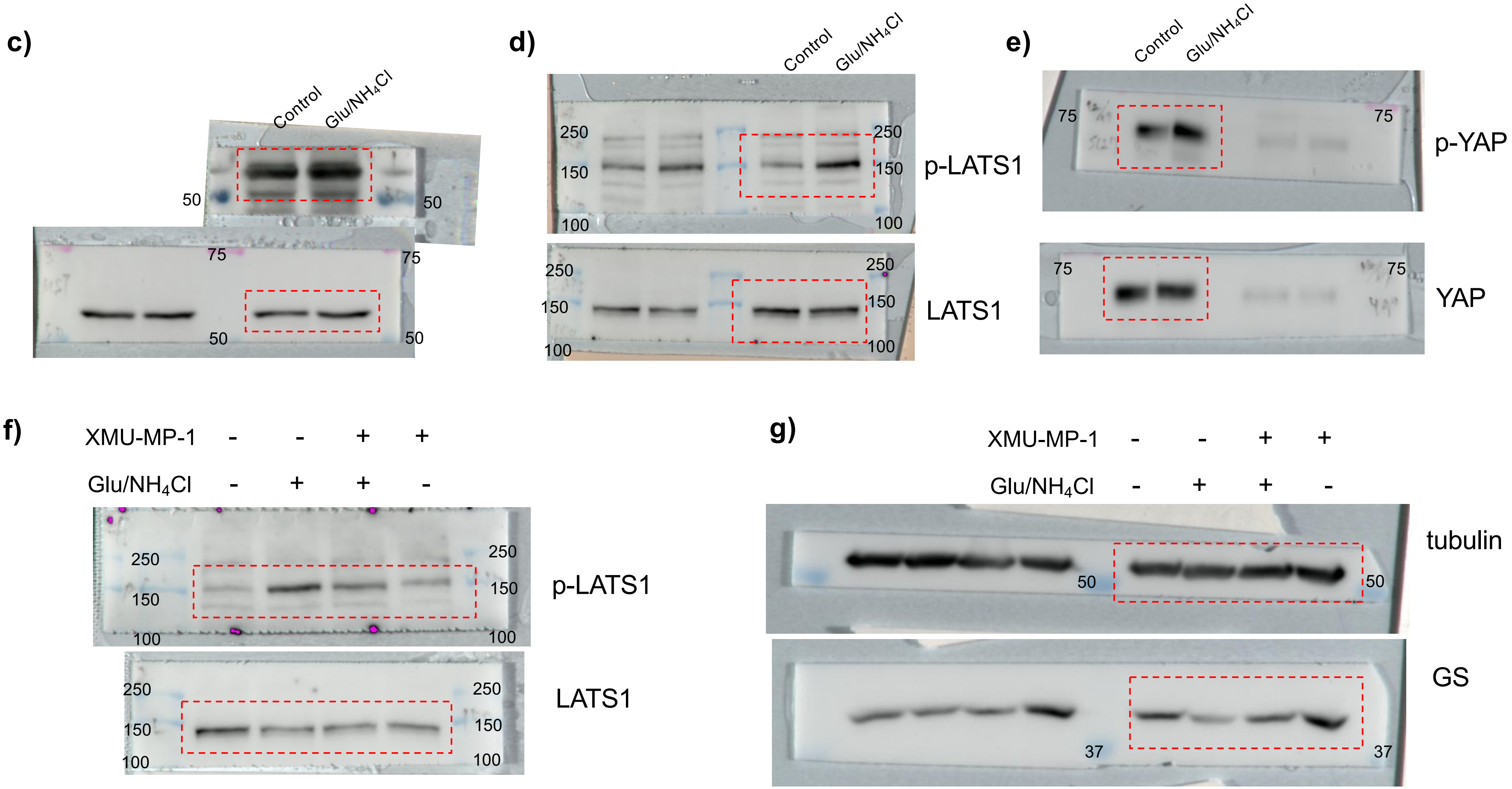
c)
Control
Glu/NH4Cl
50
50
75
75
50
50
d)
Control
Glu/NH4Cl
250
250
150
150
100
100
p-LATS1
250
250
150
150
100
100
LATS1
e)
Control
Glu/NH4Cl
75
75
p-YAP
75
75
YAP
f)
XMU-MP-1 - - + +
Glu/NH4Cl - + + -
250
250
150
150
100
100
p-LATS1
250
250
150
150
100
100
LATS1
g)
XMU-MP-1 - - + +
Glu/NH4Cl - + + -
50
50
tubulin
37
37
GS
