# Supplementary material for: Ammonia reduces glutamine synthetase expression in astrocytes via activation of Hippo-YAP signaling pathways
Source: Commun Biol. 2025 Dec 13;8:1810. doi: 10.1038/s42003-025-09191-5 (PMC12728173; doi:10.1038/s42003-025-09191-5)
Supplement: Supplementary file 2 — Description of Additional Supplementary File [file 42003_2025_9191_MOESM2_ESM.pdf]

Description of Additional Supplementary Files

File name: Supplementary Data 1

Description: The source data supporting the findings of this study are available in Supplementary Data 1
